# Sun Exposure across the Life Course Significantly Modulates Early Multiple Sclerosis Clinical Course

**DOI:** 10.3389/fneur.2018.00016

**Published:** 2018-02-01

**Authors:** Steve Simpson, Ingrid van der Mei, Robyn M. Lucas, Anne-Louise Ponsonby, Simon Broadley, Leigh Blizzard, Ausimmune/AusLong Investigators Group, Robyn M. Lucas, Bruce Taylor

**Affiliations:** ^1^Menzies Institute for Medical Research, University of Tasmania, Hobart, TAS, Australia; ^2^Melbourne School of Population and Global Health, University of Melbourne, Melbourne, VIC, Australia; ^3^National Centre for Epidemiology and Population Health, Research School of Population Health, College of Health and Medicine, Australian National University, Canberra, ACT, Australia; ^4^Centre for Ophthalmology and Visual Science, University of Western Australia, Perth, Australia; ^5^Murdoch Children’s Research Institute, Melbourne, VIC, Australia; ^6^Royal Melbourne Hospital, School of Medicine, University of Melbourne, Melbourne, VIC, Australia; ^7^School of Medicine, Griffith University, Gold Coast, QLD, Australia

**Keywords:** multiple sclerosis, vitamin D, sun exposure, ultraviolet radiation, relapse, behaviour change, first demyelinating event, CIS

## Abstract

**Background:**

Low vitamin D and/or sun exposure have been associated with increased risk of multiple sclerosis (MS) onset. However, comparatively, few studies have prospectively examined associations between these factors and clinical course.

**Objectives:**

To evaluate the association of sun exposure parameters and vitamin D levels with conversion to MS and relapse risk in a prospectively monitored cohort of 145 participants followed after a first demyelinating event up to 5-year review (AusLong Study).

**Methods:**

Sun exposure prior to and after onset measured by annual questionnaire; ultraviolet radiation (UVR) “load” estimated by location of residence over the life course and ambient UVR levels. Serum 25-hydroxyvitamin D [25(OH)D] concentrations measured at baseline, 2/3-year, and 5-year review. MS conversion and relapse assessed by neurologist assessment and medical record review.

**Results:**

Over two-thirds (69%) of those followed to 5-year review (100/145) converted to MS, with a total of 252 relapses. Higher pre-MS onset sun exposure was associated with reduced risk of MS conversion, with internal consistency between measures and dose–response relationships. Analogous associations were also seen with risk of relapse, albeit less strong. No consistent associations were observed between postonset sun exposure and clinical course, however. Notably, those who increased their sun exposure during follow-up had significantly reduced hazards of MS conversion and relapse. Serum 25(OH)D levels and vitamin D supplementation were not associated with conversion to MS or relapse hazard.

**Conclusion:**

We found that preonset sun exposure was protective against subsequent conversion to MS and relapses. While consistent associations between postonset sun exposure or serum 25(OH)D level and clinical course were not evident, possibly masked by behavior change, those participants who markedly increased their sun exposure demonstrated a reduced MS conversion and relapse hazard, suggesting beneficial effects of sun exposure on clinical course.

## Introduction

Of the various environmental and behavioral—and thus potentially modifiable—risk factors implicated in the onset and progression of multiple sclerosis (MS), evidence for a role for vitamin D and ultraviolet radiation (UVR) exposure has been among the most consistent, on a par with smoking and Epstein-Barr virus exposure. As we have discussed elsewhere (1), vitamin D and UVR exposure provide an excellent example of epidemiological consistency with both onset (2–15) and clinical course (12, 16–25), being supported by measures of preonset time in the sun, vitamin D intake, and direct measurement using measures of current sun exposure and vitamin D metabolites, and even indirectly by the well-recognized latitudinal gradient in MS prevalence and incidence (26, 27). Additional evidence comes from genetic studies that have detected significant associations with polymorphisms within or near vitamin D-associated genes (28), as well as by recent Mendelian randomization studies that implicated genetic differences in 25(OH)D levels in the development of MS (29, 30).

All this taken together has led us to wait with anticipation for the results of UV treatment and vitamin D supplementation as interventions against MS outcomes in randomized trials (31). However, the results published thus far have had inconsistent findings. Some smaller studies showed some benefit of vitamin D supplementation, either alone or in addition to established immunomodulatory therapy (32, 33), but others have shown no effects with some or all outcomes (32, 34–38). Results from larger trials are yet forthcoming, including the PrevANZ study of vitamin D supplementation as an intervention against progression to clinically definite MS following a first demyelinating event (FDE), as well as the larger SOLAR study investigating vitamin D supplementation as an add-on to interferon-beta therapy (31). Additionally, the PhoCIS Study trial of UVR phototherapy as an intervention in patients with clinically isolated syndrome (39) recently reported its preliminary results, indicating a 30% reduction in subsequent conversion to MS in the treated vs. untreated group (40).

The disparity between the relative consistency of the observational studies of the past two decades, mainly focused on MS onset, and these first randomized trials, mainly focused on clinical course, has been perplexing. One suggestion has been that sun exposure has benefits beyond vitamin D (10, 41); and therefore significant findings based on UVR-derived vitamin D from observational studies do not translate into effect in the vitamin D supplementation intervention studies as only the effect of vitamin D is being tested. Others have suggested that the null hypothesis—that vitamin D/UV associations in observational studies merely represent unmeasured confounding—is indicated by the results from randomized controlled trials (42, 43).

To better understand the roles of sun/UVR exposure and vitamin D in MS disease activity and progression, we analyzed sun exposure before and after onset of MS, as well as serum 25(OH)D levels and vitamin D supplementation after onset, for their associations with clinical outcomes in a prospective cohort study of participants followed since FDE.

## Materials and Methods

### Participants and Setting

The Ausimmune Study is a population-based multicentre case-control study designed to capture all incident FDE cases in four regions of eastern Australia: Brisbane city (latitude 27° South), Newcastle city and surrounds (33° South), Geelong city and the Western Districts of Victoria (37° South), and the state of Tasmania (43° South) (8). The Ausimmune Longitudinal (AusLong) Study, which built upon the original Ausimmune Study, seeks to elucidate environmental, genetic and personal risk factors for the onset and early progression of MS in case participants in the Ausimmune Study who had a first clinical diagnosis of CNS demyelination (FCD) and were thus at high risk of developing MS (44). Since 2009 the AusLong Study has followed Ausimmune Study cases (retention rate 84.6%). The ethics committee of all participating centers approved the study; all participants signed written informed consent.

The cohort for the present analysis is slightly different to the original Ausimmune case cohort (8, 45). Following review of clinical information at the 5-year review, three of the original 282 cases were identified as not having had an MS-associated FDE (one neuromyelitis optica spectrum disorder, one Susac’s syndrome, and one pineal germinoma). Additionally, three cases originally recorded as relapse-onset were reclassified to progressive-onset on the basis of a clinical course devoid of discrete events but instead steady deterioration in function more befitting a progressive disease course (46). With these adjustments, there were 260 relapse-onset cases (170 of whom had their FDE just prior to initial presentation and inclusion in the study and whom we define here as “classic FDE cases,” and 90 whose FDE had occurred at an earlier date). These participants were thus diagnosed as having MS on presentation to the study. In addition, there were 19 case participants with progressive-onset disease.

Our principal analysis was restricted to “classic FDE cases,” i.e., those participants with a relapse-onset presentation whose first known event occurred just prior to notification to, and participation, in the Ausimmune Study (*n* = 170). Accordingly, all of the clinical events that occurred for these individuals did so during the prospective follow-up period, giving us greatest confidence in the reliability of these data. The participants with relapse-onset presentation who had had an FDE more distant from their first participation in the Ausimmune Study, as well as the progressive-onset cases, were not included in this analysis.

### Exposure Measures

Preonset sun exposure behaviors were queried at baseline, including time spent in the sun in various age categories were queried by questionnaire and life calendar: sun exposure during 6–10, 11–15, and 16–20 years periods, and 1, 2, and 3 years prior to baseline review were specifically queried by questionnaire. In addition, summer and winter sun exposure from age 6 onward were queried by personal work and residence calendar, including time spent outside and the location of residence. From the life calendar, we used satellite data to estimate the average daily dose of UVR for each month over each year of life according to the latitude and longitude of the location of residence. We combined this with data on time outdoors in each year of life to calculate the total summer and winter UVR exposures for each individual for various periods of life, hereafter referred to as UVR-load. Postonset sun exposure for each year of follow-up in summer and winter was queried at each review (queried as <1, 1–2, 2–3, 3–4, and 4+ h/day and coded as 0, 1, 2, 3, and 4).

Change in sun exposure between baseline and 5-year review was calculated for summer and winter, and for weekdays, weekends, and holidays. Decreases of −4, −3, or −2 correspond to moving 4, 3, or 2 increments down in the sun exposure parameter between reviews, and reciprocally, +2, +3, or +4 correspond to moving 2, 3, or 4 increments up in the sun exposure parameter between reviews. Values of 0, as well as +1 and −1, were interpreted as the participant’s sun exposure for that parameter being unchanged between reviews, for example 1–2 h/day at both time points.

Serum was taken at each of the three face-to-face reviews (baseline, 2/3-year, 5-year), from which serum 25-hydroxyvitamin D_3_ (25(OH)D) levels were measured. Baseline serum 25(OH)D levels were measured at RDDT in Victoria, Australia, whereas 2/3 and 5-year serum 25(OH)D levels were measured at Canterbury Labs in New Zealand. The 25(OH)D levels from these separate assays were compared in a subset of 50 samples that were measured with both assays and also at a laboratory at the US Centers for Disease Control and Prevention (CDC) that uses a 25(OH)D assay that is standardized to the NIST-Ghent reference measurement procedure. Based on these data we calculated a conversion algorithm to standardize the 25(OH)D values from each of the assays to the CDC assay using Deming regression. The (study baseline) RMIT translation function was

Standard 25(OH)D =((As-measured 25(OH)D)×0.87)−1.74.

And for the samples assayed at Canterbury Laboratories:

Standard 25(OH)D =((As-measured 25(OH)D)×0.79)+5.91.

More comprehensive discussion of the methods involved in 25(OH)D measurement and consolidation of metrics to a standard are described elsewhere (47).

Having standardized the 25(OH)D levels to the reference laboratory for all samples at the three measurement time points, deseasonalized 25(OH)D levels were estimated using separate sinusoidal regression functions for each of the four study sites [given the widely disparate levels of ambient UVR and thus seasonal patterns of variation in 25(OH)D], using functions described previously (17). We estimated serum 25(OH)D levels at November 19 and May 20, the start of the summer and winter 6-month seasons.

### Clinical Course Measures

Two clinical outcomes were evaluated, namely hazard of conversion to MS and hazard of relapse. Conversion to MS was defined primarily as the occurrence of two or more clinical demyelinating episodes, thus satisfying the diagnostic requirements of dissemination in space and time, or a single episode plus paraclinical evidence, as per the 2005 McDonald criteria (48) including application of MRI criteria. Conversion to MS was reported at annual review and cross-checked with neurological records and confirmed by a study neurologist at 2/3 and 5 years. A relapse was defined according to the 2001 McDonald Criteria (49) as the acute or subacute appearance or reappearance of a neurological abnormality (lasting at least 24 h) in the absence of other potential explanatory factors. Relapses were reported at annual review and only relapses which were diagnosed and verified by a neurologist were included in the analysis.

### Data Analysis

Interreview differences in the proportions with different levels of the various sun/vitamin D-related covariates were assessed by Chi-square test, and thence by log-multinomial regression to determine the magnitude and significance of changes in each level of the covariates between reviews.

Predictors of hazard of MS conversion and of relapse were evaluated by Cox proportional hazards regression models, the latter for repeated events. Time at risk for both outcomes was defined as starting from the date of symptom onset. All covariates satisfied the proportional hazards assumption except that of study site for relapse analyses.

Time-varying covariates were extrapolated forwards in time from their measure as appropriate. Thus, while baseline-measured covariates and preonset parameters such as preonset sun exposure behaviors were extrapolated forward to cover all events, time-varying behavioral covariates (e.g., longitudinal sun exposure and sun behavior) were extrapolated forward only to the next annual measure of that variable, whereas serum 25(OH)D level was extrapolated forward only to 6 months from when the sample was taken, reflecting the greater variability of this parameter.

Tests for trend of categorical variables were undertaken by replacing the binary predictors with a single predictor, taking category rank scores.

All statistical analyses were conducted in Stata/SE 14.1 (StataCorp LP, College Station, TX, USA).

## Results

The cohort comprised 170 persons; however, for the purposes of evaluating change in behavior, analysis was restricted to the 145/170 persons who completed the 5-year review. There were no material differences between the total cohort and those completing the 5-year review (see Table 1), except for the proportion converting to MS and the number of recorded relapses, which were higher among those with follow-up to 5 years. During follow-up, 110 of 170 participants converted to clinically definite MS (64.7%) and experienced 265 relapses. Of the 145 participants who completed the 5-year review, 100 (69.0%) had converted to MS and between them had 252 relapses; 35 participants (24.1%) had no relapses during follow-up.

**Table 1:** Cohort characteristics, all persons and those who completed up to 5-year review.

	All persons	Completed 5-year review	Test for difference

	*n* (%)	*n* (%)	
**Sex**			

Male Female	38 (22.4) 132 (77.7)	31 (21.4) 114 (78.6)	*p* = 0.46

**Study site**			

QLD NSW VIC TAS	49 (28.8) 26 (15.3) 33 (19.4) 62 (36.5)	38 (26.2) 24 (16.6) 29 (20.0) 54 (37.2)	*p* = 0.30

**BMI at baseline^a^**			

Normal Overweight Obese	78 (46.4) 46 (27.4) 44 (26.2)	65 (44.8) 41 (28.3) 39 (26.9)	*p* = 0.58

**Diagnosed with MS during follow-up?**			

No Yes	60 (35.3) 110 (64.7)	45 (31.0) 100 (69.0)	***p* = 0.005**

**Number of relapses during study period^b^**			

0 1 2 3 4–23	60 (35.3) 59 (34.7) 20 (11.8) 11 (6.5) 20 (11.8)	45 (31.0) 51 (35.2) 19 (13.1) 10 (6.9) 20 (13.8)	***p* = 0.015**

**Taking immunomodulatory medication at 5-year review?**			

No Yes		72 (49.7%) 73 (50.3%)	

	**Mean (SD; range)**		

Age (years)	37.6 (9.7; 18–58)	37.7 (9.6; 18–58)	*p* = 0.73
As-measured serum 25(OH)D at baseline review (nmol/L)^b^	64.8 (28.9; 12.8–178.1)	63.9 (27.6; 12.8–142.5)	*p* = 0.39
Deseasonalized serum 25(OH)D at baseline review (nmol/L)^b^	64.7 (28.1; 7.9–173.7)	65.3 (28.3; 7.9–173.7)	*p* = 0.46
As-measured serum 25(OH)D at 5-year review (nmol/L)^c^		69.9 (26.5; 24.9–167.1)	
Deseasonalized serum 25(OH)D at 5-year review (nmol/L)^c^		70.3 (26.2; 21.8–169.0)	

EDSS at 5-year review,^d^ median (IQR)		1.5 (1.0–2.0)	

*Test for difference in dichotomous or categorical variables between groups assessed by Chi-square test. Test for difference in normal continuous variables [serum 25(OH)D] assessed by Student’s t-test*.

*^a^Excludes two persons who did not have BMI measured at baseline*.

*^b^Excludes six persons who did not have serum 25(OH)D measured at baseline*.

*^c^Excludes four persons who did not have serum 25(OH)D measured at 5-year review*.

*^d^Excludes three persons who did not have EDSS measured at 5-year review*.

*QLD, Queensland study site; NSW, New South Wales study site; VIC, Victoria study site; TAS, Tasmania study site; BMI, body mass index; MS, multiple sclerosis; 25(OH)D, 25-hydroxyvitamin D; EDSS, Expanded Disability Status Scale; IQR, interquartile range*.

Of the participants completing 5-year review, over three-quarters of the participants were female and the average age at study entry was 37.7 years. The largest proportions of participants were recruited in Tasmania (37.2%) and Queensland (26.2%). The average deseasonalized serum 25(OH)D concentration at baseline was 65.3 nmol/L, increasing to 70.3 nmol/L at five-year review.

### Association between Recent and Preonset Sun Exposure and Conversion to MS and Relapse

Higher preonset UVR-load parameters, both in summer and in winter, were associated with significantly reduced hazard of MS conversion (Table 2), most associations persisting on adjustment for age, sex and study site. More recent higher UVR exposure (UVR-load in the year of the FDE, 2 years before FDE, 3 years before study entry) were also significantly associated with a reduced risk of converting to MS, persisting on adjustment.

**Table 2:** Preonset sun exposure measures and associations with hazard of MS conversion.

Summer UVR measures and hazard of MS conversion				Winter UVR measures and hazard of MS conversion			

	Failures/person-years (rate)	HR (95% CI)			Failures/person-years (rate)	HR (95% CI)	
		Univariable	Adjusted^a^			Univariable	Adjusted^a^
Summer UVR-load 6–10 years old (kJ/m^2^)				Winter UVR-load 6–10 years old (kJ/m^2^)			

0–228 >228–323 >323–383 >383–488 *Trend*:	35/83.36 (0.42) 31/138.17 (0.22) 26/123.21 (0.21) 18/103.78 (0.17)	1.00 (reference) **0.60 (0.36, 0.98)** **0.56 (0.34, 0.91)** **0.44 (0.25, 0.78)** ***p* = 0.004**	1.00 (reference) 0.73 (0.43, 1.25) 0.75 (0.42, 1.34) **0.47 (0.25, 0.86)** ***p* = 0.020**	0–31 >31–52 >52–100 >100–544 *Trend*:	33/88.78 (0.37) 33/124.15 (0.27) 22/125.50 (0.18) 22/110.09 (0.20)	1.00 (reference) 0.75 (0.46, 1.22) **0.53 (0.30, 0.92)** **0.57 (0.33, 0.96)** ***p = 0.017***	1.00 (reference) 0.87 (0.50, 1.52) 0.60 (0.32, 1.15) **0.46 (0.23, 0.92)** ***p = 0.025***

Summer UVR-load 11–15 years old (kJ/m^2^)				Winter UVR-load 11–15 years old (kJ/m^2^)			

0–229 >229–313 >313–360 >360–482 *Trend*:	35/83.15 (0.42) 30/102.36 (0.29) 29/157.05 (0.19) 16/105.96 (0.15)	1.00 (reference) 0.71 (0.43, 1.18) **0.48 (0.29, 0.79)** **0.39 (0.21, 0.72)** ***p = 0.001***	1.00 (reference) 0.86 (0.51, 1.44) 0.62 (0.35, 1.09) **0.48 (0.25, 0.90)** ***p = 0.012***	0–33 >33–53 >53–104 >104–530 *Trend*:	32/105.92 (0.30) 33/100.31 (0.33) 20/134.19 (0.15) 25/108.10 (0.23)	1.00 (reference) 1.07 (0.66, 1.74) **0.55 (0.31, 0.99)** 0.78 (0.47, 1.29) *p* = *0.093*	1.00 (reference) 1.20 (0.69, 2.09) 0.63 (0.33, 1.19) 0.72 (0.39, 1.32) *p* = *0.14*

Summer UVR-load 16–20 years old (kJ/m^2^)				Winter UVR-load 16–20 years old (kJ/m^2^)			

0–188 >188–266 >266–337 >337–491 *Trend*:	36/86.43 (0.42) 29/103.58 (0.28) 28/131.22 (0.21) 17/127.29 (0.13)	1.00 (reference) 0.67 (0.41, 1.09) **0.55 (0.33, 0.92)** **0.37 (0.21, 0.66)** ***p* < *0.001***	1.00 (reference) 0.72 (0.42, 1.23) 0.75 (0.44, 1.29) **0.52 (0.28, 0.97)** *p* = *0.054*	0–28 >28–45 >45–80 >80–556 *Trend*:	36/88.02 (0.41) 34/119.76 (0.28) 19/111.86 (0.17) 21/128.88 (0.16)	1.00 (reference) 0.73 (0.46, 1.15) **0.47 (0.26, 0.84)** **0.46 (0.27, 0.76)** ***p = 0.001***	1.00 (reference) 0.81 (0.48, 1.36) **0.52 (0.27, 1.00)** **0.48 (0.27, 0.84)** ***p = 0.008***

Summer UVR-load 21–25 years old (kJ/m^2^)				Winter UVR-load 21–26 years old (kJ/m^2^)			

0–148 >148–237 >237–329 >329–494 *Trend*:	28/74.94 (0.37) 29/88.83 (0.33) 22/113.64 (0.19) 20/154.98 (0.13)	1.00 (reference) 0.81 (0.46, 1.43) **0.54 (0.30, 0.99)** **0.38 (0.21, 0.69)** ***p = 0.001***	1.00 (reference) 0.96 (0.53, 1.74) 0.68 (0.35, 1.31) **0.50 (0.26, 0.96)** ***p = 0.020***	0–24 >24–44 >44–85 >85–285 *Trend*:	34/83.92 (0.41) 24/109.24 (0.22) 17/104.21 (0.16) 24/135.03 (0.18)	1.00 (reference) 0.60 (0.35, 1.04) **0.45 (0.25, 0.83)** **0.50 (0.30, 0.83)** ***p = 0.006***	1.00 (reference) 0.56 (0.31, 1.01) **0.46 (0.24, 0.88)** **0.47 (0.27, 0.82)** ***p = 0.010***

Summer UVR-load 26–30 years old (kJ/m^2^)				Winter UVR-load 26–30 years old (kJ/m^2^)			

0–143 >143–228 >228–329 >329–510 *Trend*:	27/65.73 (0.41) 26/86.43 (0.30) 20/102.05 (0.20) 17/148.47 (0.12)	1.00 (reference) 0.73 (0.42, 1.29) **0.53 (0.30, 0.95)** **0.32 (0.17, 0.61)** ***p* < *0.001***	1.00 (reference) 0.82 (0.44, 1.55) 0.58 (0.30, 1.10) **0.40 (0.19, 0.85)** ***p = 0.012***	0–21 >21–44 >44–70 >70–411 *Trend*:	29/80.78 (0.36) 26/104.91 (0.25) 19/93.85 (0.20) 16/123.12 (0.13)	1.00 (reference) 0.76 (0.44, 1.29) 0.62 (0.34, 1.14) **0.42 (0.23, 0.78)** ***p = 0.005***	1.00 (reference) 0.80 (0.43, 1.49) 0.69 (0.34, 1.41) **0.37 (0.19, 0.75)** ***p = 0.005***

Summer UVR-load 31–35 years old (kJ/m^2^)				Winter UVR-load 31–35 years old (kJ/m^2^)			

0–136 >136–218 >218–328 >328–465 *Trend*:	26/65.70 (0.40) 17/76.95 (0.22) 18/109.99 (0.16) 16/96.35 (0.17)	1.00 (reference) 0.59 (0.31, 1.12) **0.47 (0.25, 0.87)** **0.46 (0.25, 0.85)** ***p = 0.009***	1.00 (reference) 0.84 (0.41, 1.74) 0.79 (0.36, 1.72) 0.78 (0.37, 1.62) *p* = *0.50*	0–22 >22–37 >37–69 >69–214 *Trend*:	24/78.62 (0.31) 19/105.33 (0.18) 19/81.31 (0.23) 15/83.74 (0.18)	1.00 (reference) 0.66 (0.35, 1.23) 0.82 (0.45, 1.49) 0.67 (0.37, 1.22) *p* = *0.30*	1.00 (reference) 0.77 (0.39, 1.53) 0.92 (0.46, 1.82) 0.52 (0.26, 1.05) *p* = *0.12*

Summer UVR-load in 3 years before study entry^b^ (kJ/m^2^)				Winter UVR-load in 3 years before study entry (kJ/m^2^)			

0–80 >80–90 >90–152 >152–290 *Trend*:	28/103.52 (0.27) 32/62.88 (0.51) 29/92.00 (0.32) 20/178.92 (0.11)	1.00 (reference) 1.60 (0.92, 2.76) 1.09 (0.65, 1.80) **0.44 (0.25, 0.78)** ***p = 0.001***	1.00 (reference) 1.74 (0.92, 3.29) 1.23 (0.71, 2.13) 0.56 (0.29, 1.05) ***p = 0.019***	0–17 >17–27 >27–41 >41–125 *Trend*:	32/120.02 (0.27) 33/64.41 (0.51) 22/112.22 (0.20) 22/140.66 (0.16)	1.00 (reference) 1.63 (0.99, 2.67) 0.80 (0.46, 1.40) 0.63 (0.37, 1.05) ***p = 0.019***	1.00 (reference) 1.51 (0.83, 2.75) 0.52 (0.22, 1.19) **0.28 (0.11, 0.71)** ***p = 0.002***

UVR-load in summer in year of FDE (kJ/m^2^)				UVR-load in winter in year of FDE (kJ/m^2^)			

7–28 >28–47 >47–69 >69–96 *Trend*:	33/46.32 (0.71) 28/92.25 (0.30) 26/151.17 (0.17) 19/133.64 (0.14)	1.00 (reference) **0.52 (0.29, 0.91)** **0.32 (0.19, 0.55)** **0.26 (0.15, 0.47)** ***p* < *0.001***	1.00 (reference) 0.56 (0.31, 1.01) **0.52 (0.24, 0.75)** **0.34 (0.18, 0.64)** ***p* < *0.001***	1–5 >5–9 >9–14 >14–53 *Trend*:	38/71.14 (0.53) 23/128.92 (0.18) 23/112.29 (0.21) 22/111.03 (0.20)	1.00 (reference) **0.43 (0.26, 0.73)** **0.47 (0.27, 0.81)** **0.46 (0.28, 0.74)** ***p = 0.004***	1.00 (reference) **0.47 (0.28, 0.82)** **0.46 (0.25, 0.84)** **0.36 (0.19, 0.66)** ***p = 0.001***

UVR-load in summer 2 years before FDE (kJ/m^2^)				UVR-load in winter 2 years before FDE (kJ/m^2^)			

7–28 >28–50 >50–69 >69–96 *Trend*:	32/46.07 (0.70) 29/106.24 (0.27) 27/143.46 (0.19) 20/135.22 (0.15)	1.00 (reference) **0.49 (0.28, 0.84)** **0.36 (0.21, 0.61)** **0.29 (0.16, 0.52)** ***p* < *0.001***	1.00 (reference) **0.49 (0.28, 0.85)** **0.49 (0.28, 0.83)** **0.36 (0.19, 0.68)** ***p = 0.003***	1–5 >5–9 >9–14 >14–53 *Trend*:	40/76.92 (0.52) 23/123.15 (0.19) 25/120.84 (0.21) 20/110.08 (0.18)	1.00 (reference) **0.45 (0.27, 0.76)** **0.48 (0.29, 0.79)** **0.44 (0.26, 0.74)** ***p = 0.002***	1.00 (reference) **0.43 (0.25, 0.75)** **0.52 (0.29, 0.96)** **0.33 (0.17, 0.62)** ***p = 0.001***

*Results in bold denote statistical significance (*p* < 0.05). Results in italics are for tests of trend*.

*^a^Adjusted models for MS include adjustment for age, sex, and study site*.

*^b^Two persons did not have data on their summer UVR-load in the 3 years preceding study entry*.

For relapse (Table 3), summer UVR-load at younger ages and most winter UVR-load parameters were significantly associated with a reduced relapse hazard, though none of these were robust to adjustment for age, sex, study site, and use of immunomodulatory medication.

**Table 3:** Preonset sun exposure measures and associations with relapse hazard.

Summer UVR measures and relapse hazard				Winter UVR measures and relapse hazard			

	Failures/person-years (rate)	HR (95% CI)			Failures/person-years (rate)	HR (95% CI)	
		Univariable	Adjusted^a^			Univariable	Adjusted^a^
Summer UVR-load 6–10 years old (kJ/m^2^)				Winter UVR-load 6–10 years old (kJ/m^2^)			

0–228 >228–323 >323–383 >383–488 *Trend*:	66/235.56 (0.28) 77/262.48 (0.29) 40/220.65 (0.18) 24/173.87 (0.14)	1.00 (reference) 1.16 (0.66, 2.06) 0.87 (0.51, 1.50) **0.48 (0.28, 0.83)** ***p = 0.006***	1.00 (reference) 1.11 (0.70, 1.77) 0.92 (0.59, 1.42) **0.54 (0.34, 0.87)** ***p = 0.010***	0–31 >31–52 >52–100 >100–544 *Trend*:	59/231.23 (0.26) 51/258.75 (0.20) 47/215.54 (0.22) 50/187.05 (0.27)	1.00 (reference) 0.75 (0.46, 1.24) 0.82 (0.43, 1.55) 0.76 (0.40, 1.45) *p* = *0.44*	1.00 (reference) 0.81 (0.51, 1.27) 0.97 (0.59, 1.58) 0.83 (0.45, 1.53) *p* = *0.68*

Summer UVR-load 11–15 years old (kJ/m^2^)				Winter UVR-load 11–15 years old (kJ/m^2^)			

0–229 >229–313 >313–360 >360–482 *Trend*:	66/234.11 (0.28) 76/229.39 (0.33) 39/259.77 (0.15) 26/169.30 (0.15)	1.00 (reference) 1.22 (0.70, 2.14) 0.70 (0.43, 1.14) 0.55 (0.29, 1.08) ***p = 0.015***	1.00 (reference) 1.26 (0.78, 2.02) 0.85 (0.56, 1.30) 0.67 (0.38, 1.19) *p* = *0.072*	0–33 >33–53 >53–104 >104–530 *Trend*:	61/237.34 (0.26) 48/235.30 (0.20) 43/218.56 (0.20) 55/201.37 (0.27)	1.00 (reference) 0.73 (0.45, 1.18) 0.73 (0.39, 1.36) 0.76 (0.42, 1.38) *p* = *0.39*	1.00 (reference) 0.70 (0.45, 1.09) 0.81 (0.50, 1.30) 0.87 (0.50, 1.49) *p* = *0.76*

Summer UVR-load 16–20 years old (kJ/m^2^)				Winter UVR-load 16–20 years old (kJ/m^2^)			

0–188 >188–266 >266–337 >337–491 *Trend*:	75/232.69 (0.32) 63/222.79 (0.28) 43/244.31 (0.18) 26/192.78 (0.14)	1.00 (reference) 0.94 (0.52, 1.72) 0.70 (0.42, 1.16) 0.51 (0.24, 1.07) ***p = 0.028***	1.00 (reference) 0.95 (0.54, 1.66) 0.85 (0.53, 1.35) 0.79 (0.41, 1.51) *p* = *0.38*	0–28 >28–45 >45–80 >80–556 *Trend*:	78/239.42 (0.33) 52/254.66 (0.20) 45/192.52 (0.23) 32/205.97 (0.16)	1.00 (reference) 0.70 (0.42, 1.15) 0.73 (0.36, 1.45) **0.42 (0.24, 0.71)** ***p = 0.002***	1.00 (reference) 0.73 (0.48, 1.12) 0.82 (0.43, 1.54) **0.62 (0.39, 0.98)** *p* = *0.078*

Summer UVR-load 21–25 years old (kJ/m^2^)				Winter UVR-load 21–26 years old (kJ/m^2^)			

0–148 >148–237 >237–329 >329–494 *Trend*:	60/194.49 (0.31) 36/196.10 (0.18) 63/210.98 (0.30) 33/227.37 (0.15)	1.00 (reference) 0.66 (0.37, 1.16) 1.07 (0.59, 1.95) 0.56 (0.29, 1.09) *p* = *0.29*	1.00 (reference) 0.78 (0.51, 1.18) 1.37 (0.83, 2.26) 1.06 (0.61, 1.86) *p* = *0.36*	0–24 >24–44 >44–85 >85–285 *Trend*:	66/230.68 (0.29) 51/207.96 (0.25) 19/197.42 (0.10) 56/192.87 (0.29)	1.00 (reference) 0.96 (0.55, 1.66) **0.34 (0.17, 0.65)** 0.79 (0.45, 1.37) *p* = *0.19*	1.00 (reference) 1.16 (0.73, 1.83) **0.50 (0.27, 0.92)** 1.11 (0.68, 1.81) *p* = *0.98*

Summer UVR-load 26–30 years old (kJ/m^2^)				Winter UVR-load 26–30 years old (kJ/m^2^)			

0–143 >143–228 >228–329 >329–510 *Trend*:	50/168.47 (0.30) 43/214.71 (0.20) 43/160.88 (0.27) 25/218.57 (0.11)	1.00 (reference) 0.68 (0.40, 1.17) 0.89 (0.41, 1.91) **0.45 (0.24, 0.86)** *p* = *0.093*	1.00 (reference) 0.89 (0.51, 1.54) 1.25 (0.61, 2.56) 1.00 (0.47, 2.11) *p* = *0.69*	0–21 >21–44 >44–70 >70–411 *Trend*:	46/218.58 (0.21) 51/198.80 (0.26) 25/204.45 (0.12) 39/140.81 (0.28)	1.00 (reference) 1.22 (0.73, 2.04) **0.46 (0.26, 0.82)** 0.90 (0.44, 1.82) *p* = *0.35*	1.00 (reference) 1.45 (0.89, 2.37) 0.75 (0.42, 1.32) 1.22 (0.65, 2.29) *p* = *0.87*

Summer UVR-load 31–35 years old (kJ/m^2^)				Winter UVR-load 31–35 years old (kJ/m^2^)			

0–136 >136–218 >218–328 >328–465 *Trend*:	37/156.09 (0.24) 22/165.80 (0.13) 43/187.47 (0.23) 21/149.85 (0.14)	1.00 (reference) 0.53 (0.27, 1.05) 0.99 (0.46, 2.10) 0.68 (0.35, 1.31) *p* = *0.72*	1.00 (reference) 0.71 (0.39, 1.27) 1.36 (0.69, 2.67) 1.49 (0.69, 3.21) *p* = *0.20*	0–22 >22–37 >37–69 >69–214 *Trend*:	34/200.37 (0.17) 25/167.84 (0.15) 32/163.86 (0.20) 32/127.14 (0.25)	1.00 (reference) 0.98 (0.52, 1.84) 0.92 (0.49, 1.72) 0.97 (0.42, 2.23) *p* = *0.89*	1.00 (reference) 1.20 (0.66, 2.17) 1.18 (0.61, 2.27) 1.75 (0.75, 4.10) *p* = *0.26*

Summer UVR-load in 3 years before study entry^b^ (kJ/m^2^)				Winter UVR-load in 3 years before study entry (kJ/m^2^)			

0–80 >80–90 >90–152 >152–290 *Trend*:	35/214.68 (0.16) 92/209.96 (0.44) 42/201.44 (0.21) 35/252.72 (0.14)	1.00 (reference) **2.28 (1.31, 3.97)** 1.24 (0.75, 2.03) 0.87 (0.44, 1.72) *p* = *0.14*	1.00 (reference) **2.43 (1.50, 3.95)** 1.31 (0.83, 2.08) 1.26 (0.65, 2.45) *p* = *0.77*	0–17 >17–27 >27–41 >41–125 *Trend*:	54/247.55 (0.22) 49/210.32 (0.23) 52/202.49 (0.26) 49/218.43 (0.22)	1.00 (reference) 0.70 (0.40, 1.23) 0.80 (0.36, 1.77) 0.62 (0.25, 1.51) *p* = *0.36*	1.00 (reference) 0.94 (0.61, 1.45) 1.15 (0.53, 2.46) 1.05 (0.43, 2.58) *p* = *0.89*

UVR-load in summer in year of FDE (kJ/m^2^)				UVR-load in winter in year of FDE (kJ/m^2^)			

7–28 >28–47 >47–69 >69–96 *Trend*:	55/198.76 (0.28) 51/214.40 (0.24) 66/249.43 (0.27) 28/189.82 (0.15)	1.00 (reference) 0.89 (0.52, 1.54) 1.08 (0.61, 1.90) 0.60 (0.34, 1.07) *p* = *0.23*	1.00 (reference) 1.02 (0.68, 1.53) 1.43 (0.86, 2.38) 1.05 (0.58, 1.91) *p* = *0.42*	1–5 >5–9 >9–14 >14–53 *Trend*:	72/232.12 (0.31) 32/220.90 (0.15) 39/210.57 (0.19) 57/188.82 (0.30)	1.00 (reference) **0.48 (0.29, 0.81)** 0.54 (0.27, 1.09) 0.69 (0.38, 1.25) *p* = *0.25*	1.00 (reference) 0.65 (0.42, 1.02) 0.83 (0.43, 1.59) 1.17 (0.66, 2.09) *p* = *0.62*

UVR-load in summer 2 years before FDE (kJ/m^2^)				UVR-load in winter 2 years before FDE (kJ/m^2^)			

7–28 >28–50 >50–69 >69–96 *Trend*:	55/193.88 (0.28) 64/227.79 (0.28) 52/241.93 (0.22) 33/206.86 (0.16)	1.00 (reference) 1.00 (0.56, 1.77) 0.88 (0.52, 1.49) 0.63 (0.35, 1.15) *p* = *0.12*	1.00 (reference) 1.10 (0.68, 1.78) 1.14 (0.73, 1.77) 0.95 (0.55, 1.63) *p* = *0.96*	1–5 >5–9 >9–14 >14–53 *Trend*:	64/251.92 (0.25) 39/212.00 (0.18) 39/21.68 (0.18) 62/184.86 (0.34)	1.00 (reference) 0.73 (0.41, 1.32) 0.69 (0.35, 1.34) 1.04 (0.58, 1.85) *p* = *0.90*	1.00 (reference) 0.92 (0.58, 1.45) 0.96 (0.52, 1.77) 1.43 (0.83, 2.46) *p* = *0.26*

*Results in bold denote statistical significance (*p* < 0.05). Results in italics are for tests of trend*.

*^a^Adjusted models for relapse include adjustment for age, sex, and immunomodulatory therapy, and stratified by study site*.

*^b^Two persons did not have data on their summer UVR-load in the 3 years preceding study entry*.

Analogous results were seen for absolute durations of sun exposure (data not shown).

### Association between Post-FDE Sun Exposure and Conversion to MS and Relapse

In contrast to the associations of preonset and peri-FDE sun exposure parameters with MS and relapse, post-FDE sun exposure (duration and derived UVR-load) showed no consistent associations with hazard of MS conversion or relapse. For hazard of MS conversion (Table 4), whereas there was evidence of an inverse association with weekend summer UVR-load, there was no association with weekday or holiday summer UVR-load. Near-significant trends for winter weekend and holiday time in the sun showed no such trends when ambient UVR was accounted for in UVR-load parameters. In analyses of relapse (Table 5), whereas some summer and winter time in the sun variables showed inverse associations with relapse hazard, these were largely not robust to adjustment.

**Table 4:** Postonset longitudinal sun exposure measures and associations with hazard of MS conversion.

Summer UVR measures and hazard of MS conversion				Winter UVR measures and hazard of MS conversion			

	Failures/person-years (rate)	HR (95% CI)			Failures/person-years (rate)	HR (95% CI)	
		Univariable	Adjusted^a^			Univariable	Adjusted^a^
Time in sun during week—summer (h/day)				Time in sun during week—winter (h/day)			

<1 1 to <2 2 to <3 3 to <4 4+ *Trend*:	22/115.98 (0.19) 20/114.50 (0.18) 10/51.99 (0.19) 2/22.04 (0.09) 5/68.07 (0.07)	1.00 (reference) 0.96 (0.52, 1.78) 1.02 (0.48, 2.17) 0.48 (0.10, 2.28) 0.41 (0.15, 1.13) *p* = *0.064*	1.00 (reference) 1.10 (0.56, 2.14) 1.15 (0.49, 2.67) 0.66 (0.13, 3.32) 0.55 (0.18, 1.67) *p* = *0.31*	<1 1 to < 2 2 to < 3 3 to < 4 4+ *Trend*:	36/185.55 (0.19) 13/93.23 (0.14) 6/22.40 (0.27) 1/18.22 (0.06) 3/53.18 (0.06)	1.00 (reference) 0.76 (0.40, 1.46) 1.28 (0.53, 3.11) 0.26 (0.03, 1.90) 0.32 (0.09, 1.06) ***p = 0.038***	1.00 (reference) 0.86 (0.42, 1.73) 1.43 (0.56, 3.62) 0.31 (0.04, 2.48) 0.39 (0.10, 1.51) *p* = *0.21*

Summer UVR-load during weekdays (kJ/m^2^)				Winter UVR-load during weekdays (kJ/m^2^)			

12–41 >41–62 >62–134 >134–422 *Trend*:	22/107.06 (0.21) 13/62.76 (0.21) 12/92.97 (0.13) 11/85.57 (0.13)	1.00 (reference) 1.17 (0.60, 2.27) 0.70 (0.35, 1.42) 0.91 (0.36, 2.27) *p* = *0.49*	1.00 (reference) 1.07 (0.55, 2.09) 0.79 (0.36, 1.72) 0.99 (0.36, 2.74) *p* = *0.79*	0–7 >7–17 >17–26 >26–198 *Trend*:	26/132.05 (0.20) 11/59.33 (0.19) 1180.47 (0.14) 10/76.51 (0.13)	1.00 (reference) 1.01 (0.50, 2.05) 0.88 (0.42, 1.85) 0.83 (0.37, 1.86) *p* = *0.61*	1.00 (reference) 0.98 (0.46, 2.09) 1.09 (0.48, 2.51) 0.93 (0.36, 2.39) *p* = *0.93*

Time in sun during weekend—summer (h/day)				Time in sun during weekend—winter (h/day)			

<1 1 to <2 2 to <3 3 to <4 4+ *Trend*:	6/23.58 (0.25) 18/91.84 (0.20) 15/91.78 (0.16) 12/65.02 (0.19) 8/100.35 (0.08)	1.00 (reference) 0.85 (0.34, 2.10) 0.68 (0.27, 1.71) 0.78 (0.30, 2.06) 0.35 (0.12, 1.02) ***p = 0.032***	1.00 (reference) 0.95 (0.36, 2.52) 0.75 (0.28, 2.03) 0.87 (0.31, 2.47) 0.49 (0.15, 1.52) *p* = *0.18*	<1 1 to <2 2 to <3 3 to <4 4+ *Trend*:	16/61.53 (0.26) 27/145.15 (0.19) 7/65.20 (0.11) 7/41.15 (0.17) 2/59.55 (0.03)	1.00 (reference) 0.73 (0.38, 1.39) 0.43 (0.17, 1.07) 0.65 (0.26, 1.62) **0.15 (0.03, 0.68)** ***p = 0.006***	1.00 (reference) 0.76 (0.39, 1.49) 0.40 (0.16, 1.01) 0.73 (0.28, 1.92) 0.19 (0.03, 1.05) ***p = 0.037***

Summer UVR-load during weekend (kJ/m^2^)				Winter UVR-load during weekends (kJ/m^2^)			

5–19 >19–50 >50–90 >90–176 *Trend*:	13/67.09 (0.19) 25/122.38 (0.20) 12/67.58 (0.18) 8/91.31 (0.09)	1.00 (reference) 0.98 (0.49, 1.95) 1.02 (0.43, 2.42) 0.56 (0.18, 1.73) *p* = *0.36*	1.00 (reference) 1.12 (0.53, 2.35) 0.99 (0.39, 2.51) 0.62 (0.18, 2.16) *p* = *0.49*	0–4 >4–9 >9–20 >20–121 *Trend*:	21/83.37 (0.25) 15/119.87 (0.13) 11/66.67 (0.17) 11/78.45 (0.14)	1.00 (reference) 0.54 (0.28, 1.03) 0.77 (0.37, 1.64) 0.83 (0.35, 2.00) *p* = *0.66*	1.00 (reference) 0.53 (0.26, 1.08) 0.78 (0.33, 1.82) 0.73 (0.24, 2.26) *p* = *0.63*

Time in sun during holidays—summer (h/day)				Time in sun during holidays—winter (h/day)			

<1 1 to <2 2 to <3 3 to <4 4+ *Trend*:	5/18.33 (0.27) 9/66.27 (0.14) 20/86.00 (0.23) 10/55.00 (0.18) 14/143.03 (0.10)	1.00 (reference) 0.57 (0.20, 1.68) 0.99 (0.37, 2.60) 0.72 (0.26, 1.96) 0.42 (0.15, 1.18) *p* = *0.083*	1.00 (reference) 0.87 (0.24, 3.14) 1.51 (0.49, 4.66) 1.02 (0.36, 2.91) 0.65 (0.22, 1.95) *p* = *0.26*	<1 1 to <2 2 to <3 3 to <4 4+ *Trend*:	10/43.49 (0.23) 21/124.83 (0.17) 13/69.93 (0.19) 8/51.36 (0.16) 6/75.74 (0.08)	1.00 (reference) 0.70 (0.34, 1.46) 0.85 (0.38, 1.92) 0.61 (0.24, 1.54) 0.37 (0.13, 1.06) *p* = *0.076*	1.00 (reference) 0.71 (0.33, 1.52) 0.93 (0.40, 2.16) 0.68 (0.26, 1.76) 0.48 (0.15, 1.54) *p* = *0.32*

Summer UVR-load during holidays (kJ/m^2^)				Winter UVR-load during holidays (kJ/m^2^)			

3–16 >16–31 >51–64 >64–103 *Trend*:	14/73.05 (0.19) 20/107.48 (0.19) 15/80.02 (0.19) 8/83.87 (0.10)	1.00 (reference) 0.96 (0.48, 1.92) 1.31 (0.59, 2.94) 0.75 (0.25, 2.21) *p* = *0.76*	1.00 (reference) 1.03 (0.51, 2.09) 1.52 (0.68, 3.40) 0.85 (0.27, 2.63) *p* = *0.99*	0–3 >3–6 >6–14 >14–70 *Trend*:	16/85.42 (0.19) 16/105.46 (0.15) 12/74.80 (0.16) 13/74.01 (0.18)	1.00 (reference) 0.91 (47, 1.77) 1.09 (0.49, 2.43) 1.61 (0.67, 3.87) *p* = *0.34*	1.00 (reference) 0.99 (0.51, 1.90) 1.37 (0.59, 3.15) 1.81 (0.68, 4.81) *p* = *0.22*

*Results in bold denote statistical significance (*p* < 0.05). Results in italics are for tests of trend. Two persons—one each in Queensland and Victoria—did not provide data on sun exposure durations. Three persons—one in Victoria and two in Tasmania—did not provide data on sun exposure durations during the week/weekends but did provide this for holidays. Eight persons—two in Victoria and six in New South Wales—did not provide data on sun exposures during holidays but did provide this for week/weekends*.

*^a^Adjusted models for MS conversion include adjustment for age, sex, and study site*.

**Table 5:** Postonset longitudinal sun exposure measures and associations with relapse hazard.

Summer UVR measures and relapse hazard				Winter UVR measures and relapse hazard			

	Failures/person-years (rate)	HR (95% CI)			Failures/person-years (rate)	HR (95% CI)	
		Univariable	Adjusted^a^			Univariable	Adjusted^a^
Time in sun during week—summer (h/day)				Time in sun during week—winter (h/day)			

<1 1 to <2 2 to <3 3 to <4 4+ *Trend*:	81/306.27 (0.27) 78/259.86 (0.30) 28/117.64 (0.24) 10/43.55 (0.23) 7/85.85 (0.08)	1.00 (reference) 1.37 (0.99, 1.89) 1.11 (0.63, 1.96) 1.17 (0.45, 3.05) 0.45 (0.20, 1.02) *p* = *0.23*	1.00 (reference) 1.33 (0.96, 1.85) 1.19 (0.70, 2.02) 1.35 (0.49, 3.69) 0.65 (0.28, 1.47) *p* = *0.93*	<1 1 to <2 2 to <3 3 to <4 4+ *Trend*:	123/439.51 (0.28) 58/225.86 (0.26) 16/55.34 (0.29) 4/32.60 (0.12) 3/59.87 (0.05)	1.00 (reference) 1.04 (0.75, 1.43) 1.08 (0.60, 1.94) 0.51 (0.22, 1.18) **0.23 (0.08, 0.64)** ***p = 0.013***	1.00 (reference) 1.05 (0.74, 1.48) 1.14 (0.64, 2.01) 0.70 (0.31, 1.58) 0.38 (0.14, 1.02) *p* = *0.27*

Summer UVR-load during weekdays (kJ/m^2^)				Winter UVR-load during weekdays (kJ/m^2^)			

12–41 >41–62 >62–134 >134–422 *Trend*:	60/228.55 (0.26) 52/173.53 (0.30) 45/193.22 (0.23) 46/187.89 (0.25)	1.00 (reference) 1.25 (0.83, 1.87) 1.17 (0.76, 1.81) 1.42 (0.89, 2.27) *p* = *0.17*	1.00 (reference) 1.02 (0.67, 1.55) 1.11 (0.70, 1.74) 1.24 (0.75, 2.06) *p* = *0.40*	0–7 >7–17 >17–26 >26–198 *Trend*:	64/250.24 (0.26) 39/175.02 (0.22) 56/185.92 (0.30) 44/172.02 (0.26)	1.00 (reference) 0.92 (0.55, 1.53) 1.56 (0.96, 2.54) 1.16 (0.76, 1.77) *p* = *0.18*	1.00 (reference) 0.83 (0.49, 1.40) 1.53 (0.92, 2.53) 1.10 (0.70, 1.73) *p* = *0.27*

Time in sun during weekend–summer (h/day)				Time in sun during weekend–winter (h/day)			

<1 1 to <2 2 to <3 3 to <4 4+ *Trend*:	33/125.95 (0.26) 63/222.71 (0.28) 53/180.29 (0.29) 30/127.79 (0.24) 25/156.42 (0.16)	1.00 (reference) 1.13 (0.62, 2.06) 1.23 (0.64, 2.36) 1.08 (0.60, 1.95) 0.83 (0.44, 1.56) *p* = *0.55*	1.00 (reference) 1.09 (0.63, 1.89) 1.27 (0.71, 2.28) 1.23 (0.69, 2.17) 1.14 (0.62, 2.08) *p* = *0.47*	<1 1 to <2 2 to <3 3 to <4 4+ *Trend*:	64/219.50 (0.29) 81/311.06 (0.26) 38/133.56 (0.29) 13/71.12 (0.18) 8/77.92 (0.10)	1.00 (reference) 0.93 (0.62, 1.41) 1.15 (0.75, 1.77) 0.70 (0.37, 1.31) 0.49 (0.21, 1.12) *p* = *0.12*	1.00 (reference) 0.97 (0.68, 1.39) 1.25 (0.81, 1.94) 0.99 (0.53, 1.84) 0.83 (0.41, 1.65) *p* = *0.85*

Summer UVR-load during weekend (kJ/m^2^)				Winter UVR-load during weekends (kJ/m^2^)			

5–19 >19–50 >50–90 >90–176 *Trend*:	51/206.29 (0.25) 62/224.14 (0.28) 51/166.81 (0.31) 39/185.95 (0.21)	1.00 (reference) 1.23 (0.78, 1.93) 1.47 (0.86, 2.53) 1.45 (0.84, 2.50) *p* = *0.12*	1.00 (reference) 1.35 (0.90, 2.03) 1.31 (0.77, 2.22) 1.36 (0.79, 2.34) *p* = *0.28*	0–4 >4–9 >9–20 >20–121 *Trend*:	52/206.15 (0.25) 43/241.98 (0.18) 59/172.99 (0.34) 49/162.08 (0.30)	1.00 (reference) 0.77 (0.49, 1.20) 1.49 (0.87, 2.55) 1.41 (0.89, 2.25) ***p = 0.026***	1.00 (reference) 0.83 (0.54, 1.26) 1.47 (0.83, 2.60) 1.49 (0.96, 2.33) ***p = 0.021***

Time in sun during holidays—summer (h/day)				Time in sun during holidays—winter (h/day)			

<1 1 to <2 2 to <3 3 to <4 4+ *Trend*:	35/102.50 (0.34) 53/168.81 (0.31) 44/174.09 (0.25) 39/131.26 (0.30) 32/220.96 (0.15)	1.00 (reference) 1.11 (0.61, 1.99) 0.94 (0.52, 1.70) 1.00 (0.60, 1.66) 0.63 (0.35, 1.14) *p* = *0.066*	1.00 (reference) 1.09 (0.63, 1.87) 1.07 (0.61, 1.85) 1.04 (0.65, 1.66) 0.79 (0.44, 1.41) *p* = *0.37*	<1 1 to <2 2 to <3 3 to <4 4+ *Trend*:	42/184.50 (0.23) 80/273.80 (0.29) 43/143.75 (0.30) 18/82.71 (0.22) 20/117.13 (0.17)	1.00 (reference) 1.28 (0.85, 1.93) 1.46 (0.92, 2.30) 0.97 (0.54, 1.73) 0.96 (0.45, 2.02) *p* = *0.82*	1.00 (reference) 1.18 (0.77, 1.80) 1.55 (0.97, 2.49) 1.23 (0.69, 2.18) 1.32 (0.66, 2.61) *p* = *0.21*

Summer UVR-load during holidays (kJ/m^2^)				Winter UVR-load during holidays (kJ/m^2^)			

3–16 >16–31 >51–64 >64–103 *Trend*:	57/201.21 (0.28) 59/206.06 (0.29) 52/183.31 (0.28) 34/177.07 (0.19)	1.00 (reference) 1.05 (0.72, 1.54) 1.35 (0.83, 2.19) 1.05 (0.57, 1.91) *p* = *0.56*	1.00 (reference) 1.13 (0.79, 1.61) 1.30 (0.82, 2.04) 0.97 (0.54, 1.74) *p* = *0.79*	0–3 >3–6 >6–14 >14–70 *Trend*:	49/217.59 (0.23) 50/207.88 (0.24) 43/181.93 (0.24) 60/163.08 (0.37)	1.00 (reference) 1.12 (0.74, 1.69) 1.20 (0.74, 1.95) **1.89 (1.26, 2.84)** ***p = 0.006***	1.00 (reference) 1.14 (0.78, 1.69) 1.22 (0.75, 1.97) **1.89 (1.28, 2.81)** ***p = 0.006***

*Results in bold denote statistical significance (*p* < 0.05). Results in italics are for tests of trend. Two persons—one each in Queensland and Victoria—did not provide data on sun exposure durations. Three persons—one in Victoria and two in Tasmania—did not provide data on sun exposure durations during the week/weekends but did provide this for holidays. Eight persons—two in Victoria and six in New South Wales—did not provide data on sun exposures during holidays but did provide this for week/weekends*.

*^a^Adjusted models for relapse include adjustment for age, sex, and immunomodulatory therapy, and stratified by study site*.

### Association between Serum 25(OH)D and Conversion to MS and Relapse

As shown in Table 6, neither baseline nor longitudinal serum 25(OH)D concentration, as-measured or deseasonalized, showed a significant association with risk of MS or relapse. For the main analysis, serum 25(OH)D level was carried forward as a predictor for events occurring within 6 months of the measure. Expansion forward to include events up to the next serum 25(OH)D measure did not materially change results (data not shown). Adjustment for relevant covariates did not materially change the results.

**Table 6:** As-measured and deseasonalized serum 25(OH)D associations with MS conversion and relapse hazard.

	MS conversion failures/person-years (rate)	MS conversion HR (95% CI)		Relapse failures/person-years (rate)	Relapse HR (95% CI)	
		Univariable	Adjusted^a^		Univariable	Adjusted^b^
Baseline As-measured 25(OH)D, continuous 10-unit		0.94 (0.86, 1.03) *p* = *0.21*	0.93 (0.84, 1.03) *p* = *0.17*		0.98 (0.91, 1.05) *p* = *0.56*	0.99 (0.93, 1.05) *p* = *0.62*
Baseline As-measured 25(OH)D						
<70 >70–217.6 *Trend*:	39/259.81 (0.15) 21/168.16 (0.13)	1.00 (reference) 0.84 (0.50, 1.43) *p* = *0.52*	1.00 (reference) 0.83 (0.47, 1.45) *p* = *0.51*	128/526.78 (0.25) 68/346.97 (0.20)	1.00 (reference) 0.73 (0.47, 1.15) *p* = *0.17*	1.00 (reference) 0.75 (0.51, 1.10) *p* = *0.14*
As-measured 25(OH)D, continuous 10-unit		0.98 (0.87, 1.10) *p* = *0.74*	0.94 (0.83, 1.07) *p* = *0.38*		1.01 (0.92, 1.11) *p* = *0.83*	1.00 (0.92, 1.09) *p* = *0.97*
<70 >70–217.6 *Trend*:	20/109.93 (0.18) 12/84.97 (0.14)	1.00 (reference) 0.87 (0.44, 1.75) *p* = *0.70*	1.00 (reference) 0.80 (0.39, 1.65) *p* = *0.55*	40/199.67 (0.20) 30/153.11 (0.20)	1.00 (reference) 0.89 (0.50, 1.60) *p* = *0.70*	1.00 (reference) 0.87 (0.50, 1.50) *p* = *0.61*
As-measured 25(OH)D, extrapolated up to 6 months		0.95 (0.86, 1.04) *p* = *0.24*	0.93 (0.84, 1.03) *p* = *0.14*		0.98 (0.92, 1.05) *p* = *0.56*	0.98 (0.93, 1.04) *p* = *0.54*
<70 >70–217.6 *Trend*:	34/200.87 (0.17) 21/139.68 (0.15)	1.00 (reference) 0.91 (0.54, 1.51) *p* = *0.70*	1.00 (reference) 0.86 (0.50, 1.47) *p* = *0.58*	110/424.36 (0.26) 51/283.63 (0.18)	1.00 (reference) 0.69 (0.46, 1.04) *p* = *0.076*	1.00 (reference) 0.73 (0.50, 1.05) *p* = *0.090*

*Results in bold denote statistical significance (*p* < 0.05). Results in italics are for tests of trend*.

*^a^Adjusted models for MS include adjustment for age, sex and study site*.

*^b^Adjusted models for relapse include adjustment for age, sex, and immunomodulatory medication use, and stratified on study site*.

### Associations between Other Variables and Conversion to MS and Relapse

As in Table S1 in Supplementary Material, use of any vitamin D-containing supplements at baseline (at which point 23.1% were using multivitamins containing 200–400 IU vitamin D), was not associated with subsequent hazard of MS conversion, though an inverse association was evident for subsequent relapse hazard. Longitudinal supplement use—by five-year review 41.8% were using vitamin D supplements—was not associated with either MS or relapse hazard. Restriction to supplements with 1,000 IU cholecalciferol or more did not materially impact on associations with MS or relapse (data not shown).

### Association between Changes in Sun Exposure Behaviors and Conversion to MS and Relapse

To understand why postonset levels of sun/UVR exposure and 25(OH)D levels were not associated with outcomes, we examined whether people changed their behavior due to the knowledge that UVR exposure and high 25(OH)D levels may be beneficial for MS.

We estimated the change in sun/UVR exposure behavior by subtracting the baseline data from the 5-year data. On average, time in the sun was relatively stable over the five years of follow-up for most participants, with increases or decreases of only 1 category in summer and winter during the week (81.5 and 86.3%), weekend (75.3 and 88.4%), and holidays (69.2 and 76.7%). However, a moderate number of participants increased or decreased their time in the sun appreciably over time, 18–29% increasing or decreasing at least 2 increments in summer and 11–23% in winter. Examining spaghetti plots (graphical depictions of the change in a parameter over multiple time points for each individual within one plot) of these changes by study review found them to be consistent in direction over the study, only increasing or decreasing, rather than more erratic trajectories. It is these persons who greatly increased or decreased their sun exposure over the study that showed material associations with clinical course. As in Table 7, persons who increased their sun exposure between baseline and 5-year review showed significant reductions in both risk of MS and relapse over the preceding interval, whereas those who reduced their sun exposure had a greater risk of these outcomes. Utilizing UVR-load rather than durations of time in the sun produced similar results. Fully expanding the increments of change found similar associations to consolidated increments (Table S2 in Supplementary Material).

**Table 7:** Change in postonset sun exposure measures and 25(OH)D and associations with MS conversion and relapse hazard.

	MS conversion failures/person-years (rate)	MS conversion HR (95% CI)		Relapse failures/person-years (rate)	Relapse HR (95% CI)	
		Univariable	Adjusted^a^		Univariable	Adjusted^b^
**Total study change in time in sun during week—summer (h/day)**						

−4, −2 h/day −1, 0, +1 h/day +2, + 4 h/day *Trend*:	7/30.45 (0.23) 49/319.31 (0.15) 1/33.57 (0.03)	1.47 (0.77, 2.82) 1.00 (reference) 0.21 (0.03, 1.31) ***p = 0.011***	1.62 (0.76, 3.45) 1.00 (reference) 0.24 (0.04, 1.33) ***p = 0.020***	20/65.96 (0.30) 172/667.91 (0.26) 11/90.01 (0.12)	1.01 (0.66, 1.55) 1.00 (reference) 0.52 (0.24, 1.12) *p* = *0.088*	1.13 (0.70, 1.83) 1.00 (reference) **0.46 (0.23, 0.92)** ***p = 0.029***

**Total study change in summer weekday UVR-load (kJ/m^2^)**						

−2 UVR load increments −1, 0, +1 UVR load increments +2, +3 UVR load increments *Trend*:	2/1.32 (1.51) 36/212.5 (0.17) 17/132.16 (0.13)	**7.47 (4.01, 13.89)** 1.00 (reference) 0.76 (0.42, 1.37) *p* = *0.15*	**7.24 (2.93, 17.89)** 1.00 (reference) 0.63 (0.33, 1.20) *p* = *0.054*	8/10.30 (0.78) 121/463.36 (0.26) 70/293.40 (0.24)	**2.16 (1.11, 4.20)** 1.00 (reference) 0.96 (0.64, 1.44) *p* = *0.43*	1.49 (0.76, 2.90) 1.00 (reference) 0.86 (0.60, 1.24) *p* = *0.20*

**Total study change in time in sun during weekend—summer (h/day)**						

−4, −2 h/day −1, 0, +1 h/day +2, +4 h/day *Trend*:	6/25.61 (0.23) 48/285.97 (0.17) 3/71.75 (0.04)	1.40 (0.56, 3.49) 1.00 (reference) **0.25 (0.08, 0.78)** ***p = 0.008***	2.07 (0.78, 5.54) 1.00 (reference) **0.21 (0.06, 0.71)** ***p = 0.001***	39/68.15 (0.57) 145/620.53 (0.23) 19/135.19 (0.14)	**2.02 (1.06, 3.84)** 1.00 (reference) 0.64 (0.35, 1.18) ***p = 0.011***	**2.23 (1.22, 4.09)** 1.00 (reference) 0.59 (0.34, 1.04) ***p = 0.002***

**Total study change in summer weekend UVR-load (kJ/m^2^)**						

−2 UVR load increments −1, 0, +1 UVR load increments +2, +3 UVR load increments *Trend*:	0 31/136.58 (0.23) 24/209.46 (0.12)	– 1.00 (reference) **0.50 (0.29, 0.85)** ***p = 0.010***	– 1.00 (reference) 0.58 (0.33, 1.02) *p* = *0.060*	2/5.50 (0.36) 113/383.79 (0.29) 84/377.77 (0.22)	0.91 (0.53, 1.56) 1.00 (reference) 0.78 (0.49, 1.23) *p* = *0.30*	**2.71 (1.42, 5.16)** 1.00 (reference) 0.92 (0.62, 1.36) *p* = *0.55*

**Total study change in time in sun during holidays—summer (h/day)**						

−4, −2 h/day −1, 0, +1 h/day +2, +4 h/day *Trend*:	10/36.72 (0.27) 44/279.28 (0.16) 3/67.33 (0.05)	1.73 (0.91, 3.30) 1.00 (reference) **0.28 (0.08, 0.98)** ***p = 0.002***	**2.24 (1.14, 4.41)** 1.00 (reference) **0.24 (0.06, 0.92)** ***p* < *0.001***	51/110.63 (0.46) 130/564.51 (0.23) 22/148.73 (0.15)	1.71 (1.00, 2.94) 1.00 (reference) 0.70 (0.36, 1.39) ***p = 0.020***	**1.75 (1.07, 2.86)** 1.00 (reference) 0.65 (0.36, 1.18) ***p = 0.004***

**Total study change in summer holiday UVR-load (kJ/m^2^)**						

−2 UVR load increments −1, 0, +1 UVR load increments +2, +3 UVR load increments *Trend*:	0 35/136.43 (0.26) 19/197.12 (0.10)	– 1.00 (reference) **0.38 (0.22, 0.66)** ***p = 0.001***	– 1.00 (reference) **0.37 (0.20, 0.67)** ***p = 0.001***	0 129/380.56 (0.34) 67/345.64 (0.19)	– 1.00 (reference) **0.62 (0.41, 0.96)** ***p = 0.033***	– 1.00 (reference) **0.65 (0.45, 0.94)** ***p = 0.021***

**Total study change in time in sun during week—winter (h/day)**						

−4, −2 h/day −1, 0, +1 h/day +2, +4 h/day *Trend*:	6/41.44 (0.15) 47/317.56 (0.15) 4/24.33 (0.16)	0.97 (0.44, 2.13) 1.00 (reference) 1.14 (0.43, 3.03) *p* = *0.81*	1.38 (0.60, 3.18) 1.00 (reference) 0.90 (0.31, 2.65) *p* = *0.51*	15/72.70 (0.21) 176/705.91 (0.25) 12/45.25 (0.27)	0.81 (0.49, 1.36) 1.00 (reference) 1.13 (0.45, 2.81) *p* = *0.48*	1.11 (0.70, 1.77) 1.00 (reference) 1.07 (0.42, 2.69) *p* = *0.93*

**Total study change in winter weekday UVR-load (kJ/m^2^)**						

−2 UVR load increments −1, 0, +1 UVR load increments +2, +3 UVR load increments *Trend*:	0 36/215.45 (0.17) 19/130.58 (0.15)	– 1.00 (reference) 0.88 (0.51, 1.52) *p* = *0.66*	– 1.00 (reference) 0.62 (0.32, 1.17) *p* = *0.14*	2/5.50 (0.36) 119/493.59 (0.24) 78/267.97 (0.29)	1.05 (0.56, 1.97) 1.00 (reference) 1.11 (0.65, 1.89) *p* = *0.71*	**2.82 (1.49, 5.36)** 1.00 (reference) 0.95 (0.57, 1.57) *p* = *0.76*

**Total study change in time in sun during weekend—winter (h/day)**						

−4, −2 h/day −1, 0, +1 h/day +2, +4 h/day *Trend*:	3/17.99 (0.17) 52/337.52 (0.15) 2/27.81 (0.07)	1.11 (0.32, 3.80) 1.00 (reference) 0.48 (0.11, 2.09) *p* = *0.36*	1.39 (0.41, 4.70) 1.00 (reference) 0.32 (0.06, 1.62) *p* = *0.099*	11/49.77 (0.22) 187/724.03 (0.26) 5/50.08 (0.10)	0.82 (0.46, 1.48) 1.00 (reference) 0.44 (0.15, 1.24) *p* = *0.33*	1.00 (0.59, 1.71) 1.00 (reference) 0.46 (0.18, 1.22) *p* = *0.16*

**Total study change in winter weekend UVR-load (kJ/m^2^)**						

−2 UVR load increments −1, 0, +1 UVR load increments +2, +3 UVR load increments *Trend*:	0 34/209.04 (0.16) 21/136.99 (0.15)	– 1.00 (reference) 0.93 (0.55, 1.56) *p* = *0.78*	– 1.00 (reference) 0.96 (0.54, 1.72) *p* = *0.90*	0 122/484.95 (0.25) 77/282.11 (0.27)	– 1.00 (reference) 0.99 (0.52, 1.86) *p* = *0.96*	– 1.00 (reference) 1.04 (0.60, 1.79) *p* = *0.90*

**Total study change in time in sun during holidays—winter (h/day)**						

−4, −2 h/day −1, 0, +1 h/day +2, +4 h/day *Trend*:	6/35.05 (0.17) 46/304.72 (0.15) 5/43.57 (0.12)	1.13 (0.49, 2.60) 1.00 (reference) 0.76 (0.28, 2.10) *p* = *0.53*	1.14 (0.47, 2.75) 1.00 (reference) 0.56 (0.17, 1.80) *p* = *0.29*	15/77.18 (0.19) 176/743.91 (0.27) 12/102.78 (0.12)	0.70 (0.42, 1.17) 1.00 (reference) **0.45 (0.23, 0.89)** *p* = *0.19*	0.87 (0.56, 1.35) 1.00 (reference) **0.50 (0.27, 0.95)** *p* = *0.087*

**Total study change in winter holiday UVR-load (kJ/m^2^)**						

−2 UVR load increments −1, 0, +1 UVR load increments +2, +3 UVR load increments *Trend*:	1/1.04 (0.96) 34/197.39 (0.17) 17/122.65 (0.14)	**4.31 (2.54, 7.32)** 1.00 (reference) 0.80 (0.45, 1.41) *p* = *0.29*	**2.18 (1.03, 4.59)** 1.00 (reference) 0.60 (0.28, 1.27) *p* = *0.10*	4/16.63 (0.24) 125/443.37 (0.28) 66/260.25 (0.25)	0.69 (0.34, 1.41) 1.00 (reference) 0.87 (0.55, 1.38) *p* = *0.68*	0.90 (0.42, 1.94) 1.00 (reference) 0.84 (0.55, 1.28) *p* = *0.43*

**Change in as-measured 25(OH)D during study—50 nmol/L cutoff**						

Low all High, then low High all Low, then high *Trend*:	7/35.37 (0.20) 7/46.92 (0.15) 32/255.83 (0.13) 14/89.85 (0.16)	1.00 (reference) 0.79 (0.31, 2.02) 0.67 (0.30, 1.49) 0.83 (0.36, 1.96) *p* = *0.68*	1.00 (reference) 0.44 (0.15, 1.26) 0.52 (0.25, 1.12) 0.59 (0.24, 1.45) *p* = *0.57*	13/84.71 (0.15) 9/71.70 (0.13) 131/511.92 (0.26) 43/205.43 (0.21)	1.00 (reference) 0.71 (0.30, 1.69) 1.43 (0.75, 2.75) 1.31 (0.64, 2.65) *p* = *0.23*	1.00 (reference) 0.76 (0.34, 1.70) 1.36 (0.76, 2.42) 1.14 (0.61, 2.13) *p* = *0.48*

**Change in as-measured 25(OH)D during study—80 nmol/L cutoff**						

Low all High, then low High all Low, then high *Trend*:	32/249.89 (0.13) 4/35.02 (0.11) 7/59.80 (0.12) 17/83.26 (0.20)	1.00 (reference) 0.93 (0.29, 2.96) 0.94 (0.45, 1.94) 1.61 (0.88, 2.95) *p* = *0.19*	1.00 (reference) 0.80 (0.23, 2.77) 0.95 (0.45, 1.98) 1.52 (0.79, 2.90) *p* = *0.30*	93/446.95 (0.21) 233/81.08 (0.28) 21/116.29 (0.18) 59/229.44 (0.26)	1.00 (reference) 1.08 (0.52, 2.26) 0.80 (0.44, 1.47) 1.28 (0.77, 2.14) *p* = *0.51*	1.00 (reference) 1.07 (0.57, 2.01) 0.74 (0.42, 1.29) 1.08 (0.70, 1.65) *p* = *0.99*

**Change in deseasonalized 25(OH)D during study—50 nmol/L cutoff**						

Low all High, then low High all Low, then high *Trend*:	6/32.84 (0.18) 7/47.73 (0.15) 32/246.29 (0.13) 15/101.11 (0.15)	1.00 (reference) 0.84 (0.31, 2.29) 0.75 (0.32, 1.78) 0.86 (0.34, 2.13) *p* = *0.77*	1.00 (reference) 0.52 (0.19, 1.47) 0.58 (0.25, 1.35) 0.52 (0.19, 1.41) *p* = *0.35*	12/73.56 (0.16) 9/72.50 (0.12) 130/505.75 (0.25) 45/221.95 (0.20)	1.00 (reference) 0.68 (0.28, 1.65) 1.38 (0.69, 2.75) 1.24 (0.60, 2.55) *p* = *0.30*	1.00 (reference) 0.78 (0.35, 1.72) 1.26 (0.69, 2.33) 1.09 (0.58, 2.05) *p* = *0.63*

**Change in deseasonalized 25(OH)D during study—80 nmol/L cut-off**						

Low all High, then low High all Low, then high *Trend*:	30/225.35 (0.13) 6/34.37 (0.18) 8/66.84 (0.12) 16/101.41 (0.16)	1.00 (reference) 1.34 (0.53, 3.37) 0.92 (0.45, 1.89) 1.21 (0.66, 2.22) *p* = *0.65*	1.00 (reference) 1.36 (0.60, 3.07) 0.83 (0.38, 1.78) 1.03 (0.52, 2.05) *p* = *0.94*	100/419.35 (0.24) 25/82.70 (0.30) 23/122.35 (0.19) 48/249.36 (0.19)	1.00 (reference) 1.04 (0.51, 2.10) 0.73 (0.41, 1.31) 0.84 (0.49, 1.45) *p* = *0.42*	1.00 (reference) 0.98 (0.55, 1.75) 0.67 (0.40, 1.11) 0.73 (0.46, 1.15) *p* = *0.11*

*Results in bold denote statistical significance (*p* < 0.05). Results in italics are for tests of trend*.

*^a^Adjusted models for MS include adjustment for age, sex, and study site*.

*^b^Adjusted models for relapse include adjustment for age, sex, and immunomodulatory medication use, and stratified on study site*.

*UVR, ultraviolet radiation; 25(OH)D, 25-hydroxyvitamin D*.

Evaluation of a total-study 25(OH)D trajectory, defined whether persons’ 25(OH)D levels stayed low or high from baseline to 5-year review, or whether they changed, utilizing two cut-points of 50 and 80 nmol/L, showed no association with MS or relapse (Table 7).

**Figure 1:**
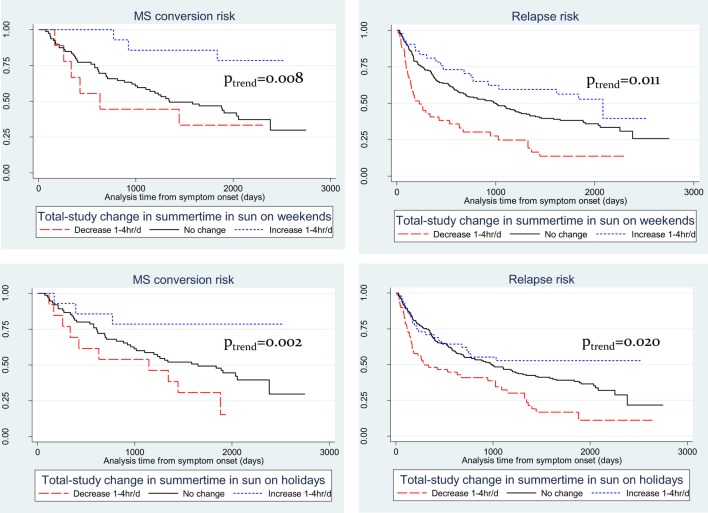
Kaplan–Meier plots of recreational summer sun exposure durations and risk of conversion to multiple sclerosis (MS) and of relapse. **(A)** summer weekend and MS; **(B)** summer weekend and relapse; **(C)** summer holidays and MS; and **(D)** summer holidays and relapse.

Viewing the results of total-study change in sun exposure behavior in Kaplan–Meier plot form (Figure 1), these trends are more evident. For MS conversion for both summer weekends (Figure 1A) and holidays (Figure 1B), whereas those who decreased their sun exposure over follow-up had no materially different conversion risk compared to those not changing sun behavior, participants who increased their sun exposure had significantly reduced hazards of MS conversion. For relapse hazard, differences were more marked for both weekend (Figure 1C) and holiday (Figure 1D), with those who decreased their sun exposure having significantly increased hazard of relapse, whereas those who increased sun exposure having significantly reduced relapse hazard.

### Changing Sun and Vitamin D-Related Behaviors during Follow-up

Given the impact of changing sun-related behaviors over the course of the study on clinical course, we next investigated when these changes in sun and related behaviors occurred.

Table 8 shows the changes that occurred over time. On average, people increased their sun and vitamin D-related behavior during follow-up, and most of the change occurred within 1–2 years after study entry. The uptake of vitamin D supplements occurred somewhat slower, but over the study duration the overall use nearly doubled from 24.1 to 40.2%, particularly vitamin D-only supplements (rather than multivitamins) where use increased 24-fold, from 1.4 to 34.8%. In parallel with this, various sun-related attire changed in favor of increased skin exposure, including the proportion wearing sunscreen in summer all the time (23.5% at 5-year from 18.3% at baseline, *p* = 0.088) or wearing short-sleeved shirts in summer all the time (69.0% at 5-year from 56.8% at baseline, *p* = 0.044), whereas the proportion reporting never wearing short-sleeved shirts in winter decreased from 89.9 to 76.6% (*p* = 0.004).

**Table 8:** Distribution of sun/vitamin D behavioral variables during study, by years poststudy entry.

	Baseline	1–1.99 years postentry	2–2.99 years postentry	3–3.99 years postentry	4–4.99 years postentry	5+ years postentry	Test for difference by Chi-square test
**Summer weekday UVR-load (kJ/m^2^)**							

12–41 >41–62 >62–134 >134–422	76 (55.9%) 24 (17.7%) 29 (21.3%) 7 (5.2%)	43 (32.6%) 23 (17.4%) 34 (25.8%)* 32 (24.2%)**	16 (12.8%) 40 (32.0%)** 30 (24.0%)** 39 (31.2%)**	15 (17.8%) 24 (22.0%)** 28 (25.7%)** 42 (38.5%)**	10 (7.8%) 28 (21.7%)** 41 (31.8%)** 50 (38.8%)**	7 (7.9%) 24 (27.0%)** 28 (31.5%)** 30 (33.7%)**	***p* < *0.001***

**Summer weekend UVR-load (kJ/m^2^)**							

5–19 >19–50 >50–90 >90–176	59 (43.4%) 63 (46.3%) 9 (6.6%) 5 (3.7%)	341 (23.5%) 47 (35.7%) 28 (21.2%)** 26 (19.7%)**	21 (16.8%) 15 (12.0%) 47 (37.6%)** 42 (33.6%)**	12 (11.0%) 21 (19.3%) 31 (28.4%)** 45 (41.3%)**	18 (14.0%) 17 (13.2%) 49 (38.0%)** 45 (34.9%)**	15 (16.9%) 8 (9.0%) 32 (36.0%)** 34 (38.2%)**	***p* < *0.001***

**Summer holiday UVR-load (kJ/m^2^)**							

3–16 >16–31 >31–64 >64–103	58 (45.0%) 56 (43.4%) 12 (9.3%) 3 (2.3%)	37 (28.5%) 41 (31.5%) 25 (19.2%)* 27 (20.8%)**	20 (16.0%) 15 (12.0%) 45 (36.0%)** 45 (36.0%)**	11 (10.2%) 19 (17.6%) 34 (31.5%)** 44 (40.7%)**	21 (16.2%) 21 (16.2%) 49 (37.7%)** 39 (30.0%)**	11 (12.2%) 16 (17.8%) 25 (27.8%)** 38 (42.2%)**	***p* < *0.001***

**Winter weekday UVR-load (kJ/m^2^)**							

0–7 >7–17 >17–26 >26–198	81 (59.6%) 26 (19.1%) 12 (8.8%) 17 (12.5%)	52 (39.4%) 20 (15.2%) 28 (21.2%)* 32 (24.2%)*	12 (9.6%) 36 (28.8%)** 41 (32.8%)** 36 (28.8%)**	14 (12.8%) 29 (26.6%)** 38 (34.9%)** 28 (25.7%)**	21 (16.3%) 31 (24.0%)** 34 (26.4%)** 43 (33.3%)**	17 (19.1%) 25 (28.1%)** 23 (25.8%)** 24 (27.0%)**	***p* < *0.001***

**Winter weekend UVR-load (kJ/m^2^)**							

0–4 >4–9 >9–20 >20–121	58 (42.7%) 55 (40.4%) 19 (14.0%) 4 (2.9%)	42 (31.8%) 41 (31.1%) 26 (19.7%) 23 (17.4%)**	21 (16.8%) 25 (20.0%) 29 (23.2%)** 50 (40.0%)**	12 (11.0%) 29 (26.6%)* 37 (33.9%)** 31 (28.4%)**	23 (17.8%) 32 (24.8%) 37 (28.7%)** 37 (28.7%)**	10 (11.2%) 20 (22.5%) 26 (29.2%)** 33 (37.1%)**	***p* < *0.001***

**Winter holiday UVR-load (kJ/m^2^)**							

0–3 >3–6 >6–14 >14–70	57 (44.5%) 44 (34.4%) 22 (17.2%) 5 (3.9%)	40 (33.1%) 35 (28.9%) 23 (19.0%)* 23 (19.0%)**	20 (17.5%) 22 (19.3%) 24 (21.1%)** 48 (42.1%)**	8 (7.9%) 30 (29.7%)** 34 (33.7%)* 29 (28.7%)**	31 (24.4%) 22 (17.3%) 39 (30.7%)** 35 (27.6%)**	12 (13.6%) 17 (19.3%) 26 (29.6%)** 33 (37.5%)**	***p* < *0.001***

**Taking vitamin D-containing supplement at review?**							

No Yes	110 (75.9%) 35 (24.1%)	115 (81.6%) 26 (18.4%)	99 (75.6%) 32 (24.4%)	73 (67.0%) 36 (33.0%)	77 (58.8%) 54 (41.2%)*	55 (59.8%) 37 (40.2%)*	***p* < *0.001***

**Taking high-dose (1,000 IU+) vitamin D-containing supplement at review?**							

No Yes	143 (98.6%) 2 (1.4%)	139 (98.6%) 2 (1.4%)	123 (93.9%) 8 (6.1%)	95 (87.2%) 14 (12.8%)*	98 (74.8%) 33 (25.2%)**	60 (65.2%) 32 (34.8%)**	***p* < *0.001***

*Restricted to persons followed up to 5-year review. Test for difference of strata by review assessed by multinomial logistic regression*.

*Results in bold denote statistical significance (p < 0.05). Results in italics are for tests of trend*.

*p < 0.05.

**p < 0.001.

*UVR, ultraviolet radiation*.

We also wanted to know whether people changed their behavior more after they converted to MS, as this is a point where patients are often being told that they have MS. For each patient, we compared the review closest to conversion with the review just after conversion of MS (Table 9). There was no consistent evidence that conversion to MS was a time point where patients changed their behavior to a large extent.

**Table 9:** Distribution of sun/vitamin D behavioral variables during study relative to MS conversion. Table restricted to those who converted to MS and who were followed up to 5-year review.

	Baseline	Postentry, prior to MS conversion	Post-MS conversion	5-Year review	Test for difference by Chi-square test
**Summer weekday UVR-load (kJ/m^2^)**					

12–41 >41–62 >62–134 >134–422	57 (59.4%) 17 (17.7%) 16 (16.7%) 6 (6.3%)	11 (18.6%) 14 (23.7%)* 20 (33.9%)** 14 (23.7%)**	50 (18.5%) 73 (26.9%)** 69 (25.5%)** 79 (29.2%)**	10 (10.0%) 31 (31.0%)** 33 (33.0%)** 26 (26.0%)**	***p* < *0.001***

**Summer weekend UVR-load (kJ/m^2^)**					

5–19 >19–50 >50–90 >90–176	46 (47.9%) 41 (42.7%) 5 (5.2%) 4 (4.2%)	7 (11.9%) 19 (32.2%)* 22 (37.3%) 11 (18.6%)	62 (22.9%) 53 (19.6%)** 85 (31.4%)** 71 (26.2%)**	20 (20.0%) 12 (12.0%)** 39 (39.0%)** 29 (29.0%)**	***p* < *0.001***

**Summer holiday UVR-load (kJ/m^2^)**					

3–16 >16–31 >31–64 >64–103	43 (47.3%) 36 (39.6%) 9 (9.9%) 3 (3.3%)	7 (11.9%) 16 (27.1%)* 26 (44.1%) 10 (17.0%)	63 (23.5%) 56 (20.9%)** 72 (26.9%)** 77 (28.7%)**	18 (18.0%) 21 (21.0%)** 33 (33.0%)** 28 (28.0%)**	***p* < *0.001***

**Winter weekday UVR-load (kJ/m^2^)**					

0–7 >7–17 >17–26 >26–198	59 (61.5%) 18 (18.8%) 7 (7.3%) 12 (12.5%)	16 (27.1%) 8 (13.6%) 22 (37.3%)** 13 (22.0%)**	53 (19.6%) 79 (29.2%)** 68 (25.1%)** 71 (26.2%)**	16 (16.0%) 27 (27.0%)** 28 (28.0%)** 29 (29.0%)**	***p* < *0.001***

**Winter weekend UVR-load (kJ/m^2^)**					

0–4 >4–9 >9–20 >20–121	46 (47.9%) 36 (37.5%) 11 (11.5%) 3 (3.1%)	11 (18.6%) 15 (25.4%) 15 (25.4%) 18 (30.5%)*	61 (22.5%) 76 (28.0%)* 73 (26.9%)** 61 (22.5%)**	12 (12.0%) 25 (25.0%)** 29 (29.0%)** 34 (34.0%)**	***p* < *0.001***

**Winter holiday UVR-load (kJ/m^2^)**					

0–3 >3–6 >6–14 >14–70	44 (48.4%) 26 (28.6%) 17 (18.7%) 4 (4.4%)	8 (15.1%) 18 (34.0%)* 9 (17.0%) 18 (34.0%)	66 (26.3%) 60 (23.9%) 64 (25.5%)* 61 (24.3%)**	16 (16.2%) 19 (19.2%)** 32 (32.3%)** 32 (32.3%)**	***p* < *0.001***

**Taking vitamin D-containing supplement at review?**					

No Yes	75 (75.0%) 25 (25.0%)	46 (74.2%) 16 (25.8%)	201 (73.1%) 74 (26.9%)	56 (56.0%) 44 (44.0%)*	***p = 0.006***

**Taking high-dose (1,000 IU +) vitamin D-containing supplement at review?**					

No Yes	98 (98.0%) 2 (2.0%)	60 (96.8%) 2 (3.2%)	243 (88.4%) 32 (11.6%)*	61 (61.0%) 39 (39.0%)**	***p* < *0.001***

*Test for difference of strata by review assessed by multinomial logistic regression*.

*Results in bold denote statistical significance (p < 0.05). Results in italics are for tests of trend*.

*p < 0.05.

**p < 0.001.

*UVR, ultraviolet radiation*.

## Discussion

In this prospective cohort study of classic FDE cases followed for five years after FDE, we have shown that preonset sun exposure, particularly during childhood and adolescence, significantly predicts a reduced hazard of subsequent disease activity in early MS, including hazards of conversion to MS and of relapse. No consistent associations were observed between postonset sun exposure or serum 25(OH)D level and clinical outcomes. Interestingly, however, we found that those who increased their sun exposure during the study had a significantly lower risk of MS conversion and relapse than those whose sun exposure decreased or remained static.

The finding that preonset sun exposure behavior going back to early childhood significantly modulates clinical outcomes years later is a novel one. There is strong and consistent evidence from case–control studies that low childhood sun exposure (2, 5, 50) or vitamin D intake (9) are associated with increased risk of later being a MS case. Using the superior longitudinal cohort study design, we have likewise found significant protective associations of childhood and adolescent sun exposure with subsequent clinical course. These associations were particularly strong for conversion to MS, though there were some weaker associations with relapse. In addition, similar associations were seen for sun exposure in the period just preceding symptom onset, substantiating a role for sun exposure across the pre-FDE life course.

Surprisingly, we found no consistent evidence of an association between post-FDE sun exposure and either MS conversion or relapse hazard, nor between postonset serum 25(OH)D levels and clinical course. These null findings were unexpected and inconsistent with other observations in support of a role for vitamin D in MS clinical course (20–24, 26, 27, 51–53). It raises the question whether there truly is no association (affirming the null hypothesis) or whether the study failed to detect an association (a false negative). Despite having the correct temporality in terms of assessing sun/vitamin D prior to the outcomes, it is possible that there were some unmeasured changes that we did not capture. For example, sun exposure and vitamin D supplementation measures were carried forward for one year, and unmeasured changes in that year could have biased the results toward the null. On average, people increased their sun and vitamin D-related behavior during the study and we found that most of the sun-related changes occurred within 1–2 years after study entry, whereas vitamin D supplementation uptake occurred somewhat slower. This may suggest a Hawthorne effect, where people enrolled in a study are inclined to change the behavior being evaluated only because it is being studied. It is possible that this limited our ability to detect an association. We examined whether conversion to MS was a timepoint where patients changed their behavior, as they were told that they have MS and may have searched for potentially beneficial lifestyle behaviors, but there was no strong evidence for this. Certainly, however, the marked increases in sun exposure and vitamin D supplementation during follow-up may have impacted on our ability to assess these parameters’ associations with clinical outcomes, since they would no longer be solely distributed by clinical phenotype.

We were unable to fully evaluate whether these findings partly reflected disease or medical-initiated alterations to these exposures over time due to the non-randomized design. Our favored interpretation is that these results indicate behavior change by participants in sun exposure/vitamin D-related parameters did occur after disease onset; this in keeping with results in this cohort seen for diet and supplementation behavior (54, 55). This interpretation is guided partly by our finding of significant behavior change in this cohort, but also by the results from our analyses of total-study change in sun behavior and its association with MS conversion and relapse hazard. In those change analyses, we found three trajectories divided by participants’ change in sun behavior. In one group, there was little or no change in UVR, of whom roughly 75% converted to MS and 60% failed by relapse. Among the subset that decreased their UVR by two or more levels, whereas their risk of MS was no different to the reference level, the proportion that failed by relapse was 80–90%, significantly greater than those whose UVR was unchanged. This result may be reflective of reverse causality – those who had greater clinical activity could realize decreased sun exposure. The other group, those who increased UVR during the study, is of interest – this group realized significantly lower proportions converting to MS (<25%) and also lower proportions failing by relapse (<50%). This is not what one would expect by reverse causality and may allude to some beneficial effect of increasing sun exposure levels on the clinical course of MS. No associations were seen for levels of 25(OH)D, changes in 25(OH)D, vitamin D supplementation nor changes in vitamin D supplementation, which could suggest that UVR interventions, such as narrow-band UVR (39), could be more promising in producing beneficial outcomes for people with MS than vitamin D supplementation alone.

Our study is unique in that it collected detailed prospective data on sun exposure, 25(OH)D levels and a large range of potential confounding variables. Though some measurement error of past sun exposure cannot be excluded, we used questions with demonstrated validity and reliability (7). The multicentre nature of the cohort across the east coast of Australia allows it to be nationally representative, and Australia is demographically and culturally similar to other European-descent populations where MS is prevalent. The prospective nature of the follow-up post-FDE with multiple time points of assessment would moderate the impact of reverse causality. The restriction to those with a classic FDE reduces the power of the study but increases the specificity of the findings. We do not have data on MRI metrics of disease activity largely due to the heterogeneous nature of the baseline scans collected (56).

In conclusion, we found that preonset sun exposure was protective against subsequent conversion to MS and relapse. While we did not find an association between postonset sun exposure or serum 25(OH)D level and clinical course, possibly due to disease or medically induced behavior change, those participants who markedly increased their sun exposure demonstrated a reduced MS conversion and relapse hazard compared to those who did not. Clinical trials investigating the effects of vitamin D supplementation and narrow-band UVR exposure will be beneficial to further substantiate the role of UVR and vitamin D in MS clinical course.

## Ausimmune/Auslong Investigators Group List

The members of the Ausimmune/AusLong Investigators Group are as follows: Robyn M. Lucas (National Centre for Epidemiology and Population Health, Canberra), Keith Dear (Duke Kunshan University, Kunshan, China), Anne-Louise Ponsonby and Terry Dwyer (Murdoch Childrens Research Institute, Melbourne, Australia), Ingrid van der Mei, Leigh Blizzard, Steve Simpson, Jr., and Bruce V. Taylor (Menzies Institute for Medical Research, University of Tasmania, Hobart, Australia), Simon Broadley (School of Medicine, Griffith University, Gold Coast Campus, Australia), Trevor Kilpatrick (Centre for Neurosciences, Department of Anatomy and Neuroscience, University of Melbourne, Melbourne, Australia), David Williams and Jeanette Lechner-Scott (University of Newcastle, Newcastle, Australia), Cameron Shaw and Caron Chapman (Barwon Health, Geelong, Australia), Alan Coulthard (University of Queensland, Brisbane, Australia), Michael P. Pender (The University of Queensland, Brisbane, Australia), and Patricia Valery (QIMR Berghofer Medical Research Institute, Brisbane, Australia).

## Ethics Statement

The ethics committee of all participating centers approved the study; all participants signed written informed consent.

## Author Contributions

SS had full access to all data in the study and takes responsibility for the integrity of the data and the accuracy of the data analysis. SS, IvdM, RL, A-LP, SB, LB, and BT as well as the total Ausimmune/AusLong Investigator Group have been involved in the conception, design, and conduct of the study. IvdM, RL, A-LP, SB, and BT provided substantial input in the drafting of the manuscript. IvdM and BT also provided feedback on the data analysis and co-wrote substantial sections of the manuscript. LB provided statistical support.

## Conflict of Interest Statement

The authors declare that the research was conducted in the absence of any commercial or financial relationships that could be construed as a potential conflict of interest.
