# Supplementary material for: Sun Exposure across the Life Course Significantly Modulates Early Multiple Sclerosis Clinical Course
Source: Front Neurol. 2018 Feb 1;9:16. doi: 10.3389/fneur.2018.00016 (PMC5799286; doi:10.3389/fneur.2018.00016)
Supplement: Supplementary file 1 [file Table_1.docx]

# Supplemental Tables

Supplementary Table 1. Other factors evaluated for associations with MS conversion & relapse hazard.

|  | MS conversion failures/person-years (rate) | MS conversion HR (95% CI) |  | Relapse failures/person-years (rate) | Relapse HR (95% CI) |  | |
| --- | --- | --- | --- | --- | --- | --- | --- |
|  |  | Univariable | Adjusted^a^ |  | Univariable | Adjusted^b^ | |
| Taking vitamin D-containing supplement at baseline? | | |  |  |  |  | |
| No  Yes | 50/345.90 (0.15)  11/84.41 (0.13) | 1.00 [Reference]  0.90 (0.46, 1.75)  *p=0.76* | 1.00 [Reference]  0.86 (0.42, 1.79)  *p=0.69* | 172/689.18 (0.25)  35/203.39 (0.17) | 1.00 [Reference]  0.69 (0.43, 1.13)  *p=0.14* | 1.00 [Reference]  **0.57 (0.36, 0.91)**  ***p=0.017*** | |
| Taking vitamin D-containing supplement at review? | | |  |  |  |  | |
| No  Yes | 46/284.82 (0.16)  13/87.76 (0.15) | 1.00 [Reference]  0.93 (0.49, 1.76)  *p=0.83* | 1.00 [Reference]  0.88 (0.46, 1.71)  *p=0.71* | 156/618.84 (0.25)  48/195.27 (0.25) | 1.00 [Reference]  0.92 (0.61, 1.38)  *p=0.68* | 1.00 [Reference]  0.87 (0.59, 1.29)  *p=0.48* | |
| Taking omega 3/fish oil-containing supplement at baseline? | | |  |  |  |  | |
| No  Yes | 55/384.92 (0.14)  6/45.39 (0.13) | 1.00 [Reference]  0.94 (0.43, 2.05)  *p=0.88* | 1.00 [Reference]  0.84 (0.34, 2.06)  *p=0.70* | 173/770.82 (0.22)  34/121.75 (0.28) | 1.00 [Reference]  1.29 (0.72, 2.33)  *p=0.40* | 1.00 [Reference]  1.09 (0.69, 1.70)  *p=0.72* | |
| Type of omega3/fish-oil-containing supplement at baseline? | | |  |  |  |  | |
| None  Fish-based  Non-fish-based | 55/384.92 (0.14)  3/36.88 (0.08)  3/8.51 (0.35) | 1.00 [Reference]  0.58 (0.25, 1.36)  2.53 (0.50, 12.88) | 1.00 [Reference]  0.52 (0.21, 1.30)  2.03 (0.24, 16.93) | 173/770.82 (0.22)  19/94.34 (0.20)  15/27.41 (0.55) | 1.00 [Reference]  0.99 (0.41, 2.39)  **1.95 (1.01, 3.77)** | 1.00 [Reference]  0.91 (0.46, 1.78)  1.41 (0.90, 2.20) | |
| Taking omega 3/fish oil-containing supplement at review? | | |  |  |  |  | |
| No  Yes | 50/317.35 (0.16)  9/55.23 (0.16) | 1.00 [Reference]  1.10 (0.55, 2.18)  *p=0.80* | 1.00 [Reference]  1.10 (0.52, 2.34)  *p=0.81* | 164/658.16 (0.25)  40/155.95 (0.26) | 1.00 [Reference]  1.04 (0.66, 1.63)  *p=0.88* | 1.00 [Reference]  0.98 (0.64, 1.49)  *p=0.91* | |
| Type of omega3/fish-oil-containing supplement at review? | | |  |  |  |  | |
| None  Fish-based  Non-fish-based | 50/317.35 (0.16)  5/32.34 (0.16)  4/22.89 (0.18) | 1.00 [Reference]  0.96 (0.40, 2.28)  1.36 (0.45, 4.07) | 1.00 [Reference]  0.98 (0.38, 2.54)  1.30 (0.42, 3.98) | 164/658.16 (0.25)  24/78.79 (0.31)  16/77.16 (0.21) | 1.00 [Reference]  1.26 (0.72, 2.19)  0.83 (0.49, 1.40) | 1.00 [Reference]  1.18 (0.72, 1.94)  0.79 (0.47, 1.34) | |
| Results in bold denote statistical significance (p<0.05). Results in italics are for tests of trend.  ^a^ Adjusted models for MS include adjustment for age, sex and study site.  ^b^ Adjusted models for relapse include adjustment for age, sex, and immunomodulatory medication use, and stratified on study site. | | | | | | |  |
