# Supplementary material for: Sun Exposure across the Life Course Significantly Modulates Early Multiple Sclerosis Clinical Course
Source: Front Neurol. 2018 Feb 1;9:16. doi: 10.3389/fneur.2018.00016 (PMC5799286; doi:10.3389/fneur.2018.00016)
Supplement: Supplementary file 2 [file Table_2.docx]

Supplementary Table 2. Change in post-onset sun exposure measures and associations with MS & relapse risk.

|  | MS failures/person-years (rate) | MS HR (95% CI) |  | Relapse failures/person-years (rate) | Relapse HR (95% CI) |  | |
| --- | --- | --- | --- | --- | --- | --- | --- |
|  |  | Univariable | Adjusted^a^ |  | Univariable | Adjusted^b^ | |
| Total study change in time in sun during week – Summer (hrs/day) | | | |  |  |  | |
| -4, -2  -1  0  +1  +2, 4^c^  *Trend:* | 7/30.45 (0.23)  18/94.14 (0.19)  19/114.59 (0.17)  12/110.59 (0.11)  1/33.57 (0.03) | 1.33 (0.62, 2.87)  1.10 (0.57, 2.10)  1.00 [Reference]  0.64 (0.30, 1.37)  0.19 (0.03, 1.23)  ***p=0.007*** | 1.23 (0.45, 3.38)  1.24 (0.63, 2.45)  1.00 [Reference]  0.65 (0.29, 1.47)  **0.16 (0.03, 0.94)**  ***p=0.010*** | 20/65.96 (0.30)  52/189.23 (0.28)  79/288.17 (0.27)  41/190.51 (0.22)  11/90.01 (0.12) | 0.98 (0.54, 1.77)  1.03 (0.59, 1.82)  1.00 [Reference]  0.82 90.43, 1.59)  0.50 (0.22, 1.13)  *p=0.085* | 1.20 (0.67, 2.18)  1.05 (0.64, 1.71)  1.00 [Reference]  0.84 (0.47, 1.50)  **0.45 (0.21, 0.96)**  ***p=0.016*** | |
| Total study change in time in sun during weekend – Summer (hrs/day) | | | |  |  |  | |
| -4, -2  -1  0  +1  +2, 4  *Trend:* | 6/25.61 (0.23)  15/65.16 (0.23)  23/145.24 (0.16)  10/75.57 (0.13)  3/71.75 (0.04) | 1.50 (0.57, 3.96)  1.49 (0.79, 2.80)  1.00 [Reference]  0.85 (0.41, 1.76)  **0.27 (0.08, 0.87)**  ***p=0.003*** | 1.90 (0.68, 5.32)  1.50 (0.75, 2.98)  1.00 [Reference]  0.77 (0.33, 1.81)  **0.26 (0.09, 0.82)**  ***p=0.001*** | 39/68.15 (0.57)  33/152.81 (0.22)  89/322.03 (0.28)  23/145.70 (0.16)  19/135.19 (0.14) | 1.76 (0.89, 3.48)  0.80 (0.50, 1.28)  1.00 [Reference]  0.58 (0.29, 1.19)  0.55 (0.28, 1.05)  ***p=0.011*** | 1.88 (1.00, 3.54)  0.67 (0.45, 1.02)  1.00 [Reference]  0.55 (0.29, 1.03)  **0.50 (0.28, 0.91)**  ***p=0.008*** | |
| Total study change in time in sun during holidays – Summer (hrs/day) | | | |  |  |  | |
| -4, -2  -1  0  +1  +2, 4  *Trend:* | 10/36.72 (0.27)  13/61.84 (0.21)  21/131.73 (0.16)  10/85.71 (0.12)  3/67.33 (0.05) | 1.72 (0.84, 3.55)  1.32 (0.67, 2.61)  1.00 [Reference]  0.73 (0.35, 1.52)  0.28 (0.08, 1.01)  ***p=0.001*** | 1.72 (0.80, 3.72)  1.14 (0.56, 2.35)  1.00 [Reference]  0.62 (0.28, 1.41)  **0.26 (0.07, 0.99)**  ***p=0.001*** | 51/110.63 (0.46)  26/12492 (0.21)  78/290.64 (0.27)  26/148.95 (0.18)  22/148.73 (0.15) | 1.50 (0.84, 2.67)  0.82 (0.46, 1.48)  1.00 [Reference]  0.66 (0.33, 1.30)  0.61 (0.30, 1.26)  ***p=0.019*** | 1.55 (0.92, 2.60)  0.83 (0.46, 1.48)  1.00 [Reference]  0.71 (0.39, 1.29)  0.59 (0.31, 1.11)  ***p=0.007*** | |
| Total study change in time in sun during week – Winter (hrs/day) | | | |  |  |  | |
| -4, -2  -1  0  +1  +2, 4  *Trend:* | 6/41.44 (0.15)  12/91.91 (0.13)  26/162.38 (0.16)  9/63.27 (0.14)  4/24.33 (0.16) | 0.89 (0.39, 2.04)  0.82 (0.41, 1.64)  1.00 [Reference]  0.89 (0.42, 1.89)  1.06 (0.39, 2.89)  *p=0.72* | 1.48 (0.57, 3.81)  0.97 (0.43, 2.21)  1.00 [Reference]  0.88 (0.38, 2.06)  0.55 (0.12, 2.53)  *p=0.33* | 15/72.70 (0.21)  46/186.45 (0.25)  97/383.79 (0.25)  33/135.67 (0.24)  12/45.25 (0.27) | 0.82 (0.45, 1.52)  1.08 (0.64, 1.83)  1.00 [Reference]  0.97 (0.48, 1.96)  1.15 (0.45, 2.94)  *p=0.78* | 1.26 (0.71, 2.21)  1.16 (0.73, 1.86)  1.00 [Reference]  1.01 (0.54, 1.88)  1.22 (0.51, 2.91)  *p=0.69* | |
| Total study change in time in sun during weekend – Winter (hrs/day) | | | |  |  |  | |
| -4, -2  -1  0  +1  +2, 4  *Trend:* | 3/17.99 (0.17)  10/85.47 (0.12)  30/186.49 (0.16)  12/65.57 (0.18)  2/27.81 (0.07) | 1.07 (0.31, 3.75)  0.74 (0.37, 1.47)  1.00 [Reference]  1.16 (0.60, 2.26)  0.46 (0.10, 2.06)  *p=0.99* | 1.40 (0.36, 5.47)  0.88 (0.43, 1.80)  1.00 [Reference]  1.10 (0.52, 2.32)  0.34 (0.05, 2.25)  *p=0.48* | 11/49.77 (0.22)  36/151.73 (0.24)  95/403.71 (0.24)  56/168.58 (0.33)  5/50.08 (0.10) | 0.91 (0.50, 1.64)  1.15 (0.46, 2.89)  1.00 [Reference]  1.30 (0.79, 2.13)  0.48 (0.17, 1.40)  *p=0.98* | 1.16 (0.69, 1.96)  1.19 (0.53, 2.66)  1.00 [Reference]  1.11 (0.72, 1.73)  0.55 (0.20, 1.51)  *p=0.50* | |
| Total study change in time in sun during holidays – Winter (hrs/day) | | | |  |  |  | |
| -4, -2  -1  0  +1  +2, 4  *Trend:* | 6/35.05 (0.17)  19/126.40 (0.15)  16/108.93 (0.15)  11/69.39 (0.16)  5/43.57 (0.12) | 1.16 (0.46, 2.93)  1.03 (0.54, 1.96)  1.00 [Reference]  1.08 (0.50, 2.33)  0.78 (0.26, 2.33)  *p=0.65* | 1.28 (0.49, 3.32)  1.07 (0.56, 2.06)  1.00 [Reference]  0.97 (0.42, 2.25)  0.50 (0.12, 2.03)  *p=0.25* | 15/77.18 (0.19)  66/225.13 (0.29)  65/.253.46 (0.26)  45/165.32 (0.27)  12/102.78 (0.12) | 0.73 (0.42, 1.27)  1.18 (0.63, 2.21)  1.00 [Reference]  0.96 (0.56, 1.66)  **0.47 (0.22, 0.99)**  *p=0.22* | 0.92 (0.56, 1.53)  1.25 (0.72, 2.19)  1.00 [Reference]  0.91 (0.53, 1.54)  0.52 (0.26, 1.04)  *p=0.063* | |
| Results in bold denote statistical significance (p<0.05). Results in italics are for tests of trend.  ^a^ Adjusted models for MS include adjustment for age, sex and study site.  ^b^ Adjusted models for relapse include adjustment for age, sex, and immunomodulatory medication use, and stratified on study site.  ^c^ Change in sun exposure presented as increments of change between quintile increments of sun exposure (summer/winter; weekday/weekend/holiday) between baseline and 5-year reviews. Thus, a decrease of -4, -3, or -2 correspond to moving 4, 3, or 2 increments down in the sun exposure parameter between reviews, and reciprocally, +2, +3 or +4 correspond to moving 2, 3, or 4 increments up in the sun exposure parameter between reviews. A value of 0 means the participant’s sun exposure for that parameter were unchanged between reviews, for example 1hr/d at both time points. | | | | | | |  |
